# Contrasting mechanisms for hidden hearing loss: Synaptopathy vs myelin defects

**DOI:** 10.1371/journal.pcbi.1008499

**Published:** 2021-01-22

**Authors:** Maral Budak, Karl Grosh, Aritra Sasmal, Gabriel Corfas, Michal Zochowski, Victoria Booth

**Affiliations:** 1 Biophysics Program, University of Michigan, Ann Arbor, Michigan, United States of America; 2 Department of Mechanical Engineering, University of Michigan, Ann Arbor, Michigan, United States of America; 3 Department of Biomedical Engineering, University of Michigan, Ann Arbor, Michigan, United States of America; 4 Kresge Hearing Research Institute, University of Michigan, Ann Arbor, Michigan, United States of America; 5 Department of Otolaryngology Head and Neck Surgery, University of Michigan, Ann Arbor, Michigan, United States of America; 6 Department of Physics, University of Michigan, Ann Arbor, Michigan, United States of America; 7 Departments of Mathematics & Anesthesiology, University of Michigan, Ann Arbor, Michigan, United States of America; University of California at Berkeley, UNITED STATES

## Abstract

Hidden hearing loss (HHL) is an auditory neuropathy characterized by normal hearing thresholds but reduced amplitudes of the sound-evoked auditory nerve compound action potential (CAP). In animal models, HHL can be caused by moderate noise exposure or aging, which induces loss of inner hair cell (IHC) synapses. In contrast, recent evidence has shown that transient loss of cochlear Schwann cells also causes permanent auditory deficits in mice with similarities to HHL. Histological analysis of the cochlea after auditory nerve remyelination showed a permanent disruption of the myelination patterns at the heminode of type I spiral ganglion neuron (SGN) peripheral terminals, suggesting that this defect could be contributing to HHL. To shed light on the mechanisms of different HHL scenarios observed in animals and to test their impact on type I SGN activity, we constructed a reduced biophysical model for a population of SGN peripheral axons whose activity is driven by a well-accepted model of cochlear sound processing. We found that the amplitudes of simulated sound-evoked SGN CAPs are lower and have greater latencies when heminodes are disorganized, i.e. they occur at different distances from the hair cell rather than at the same distance as in the normal cochlea. These results confirm that disruption of heminode positions causes desynchronization of SGN spikes leading to a loss of temporal resolution and reduction of the sound-evoked SGN CAP. Another mechanism resulting in HHL is loss of IHC synapses, i.e., synaptopathy. For comparison, we simulated synaptopathy by removing high threshold IHC-SGN synapses and found that the amplitude of simulated sound-evoked SGN CAPs decreases while latencies remain unchanged, as has been observed in noise exposed animals. Thus, model results illuminate diverse disruptions caused by synaptopathy and demyelination on neural activity in auditory processing that contribute to HHL as observed in animal models and that can contribute to perceptual deficits induced by nerve damage in humans.

## Author summary

Hidden hearing loss (HHL) is an auditory disorder caused by noise exposure, aging or peripheral neuropathy. It is named ‘hidden’ because it does not affect hearing thresholds, but deficits are observed in sound-evoked auditory nerve activity. Studies on animal models suggest two possible pathogenic mechanisms for HHL: (1) loss of synapses between inner hair cells and auditory nerve fibers, and (2) disruption of auditory-nerve myelin. In this study, we constructed a computational model of sound-evoked auditory nerve fiber activity to understand how each of these mechanisms affects nerve compound action potentials. We show that disruption of auditory-nerve myelin desynchronizes sound-evoked auditory neuron spiking, decreasing the amplitude and increasing the latency of the compound action potential. In addition, elongation of the initial axon segment may cause spike generation failure leading to decreased spiking probability. In contrast, synapse loss only decreases the probability of firing, thus reducing the compound action potential amplitude without disturbing its latency. This model, which accurately represents *in vivo* findings on HHL in the first stages of auditory processing, can be useful to analyze the consequences of synaptopathy and myelinopathy on downstream sound processing and perceptual deficits.

## Introduction

Hidden hearing loss (HHL) is defined as an auditory neuropathy characterized by changes in sound-evoked neural output of the auditory nerve (AN) without hearing threshold elevation [1]. In animal models, HHL has been directly detected by measuring AN responses to suprathreshold sound through the auditory brainstem response (ABR), a far-field response measured by head-mounted electrodes, or through the compound action potential (CAP), a near-field response measured from the round window. The first peak of the ABR (ABR peak 1) represents the activity of type I spiral ganglion neurons (SGNs) in response to sound, and the CAP reflects the synchronous response of the SGN fibers at the sound onset [1]. In humans, characterization of HHL remains inconclusive with mixed reports of presumptive AN damage resulting in perceptual deficits despite normal auditory thresholds [2–5]

**Fig 1:**
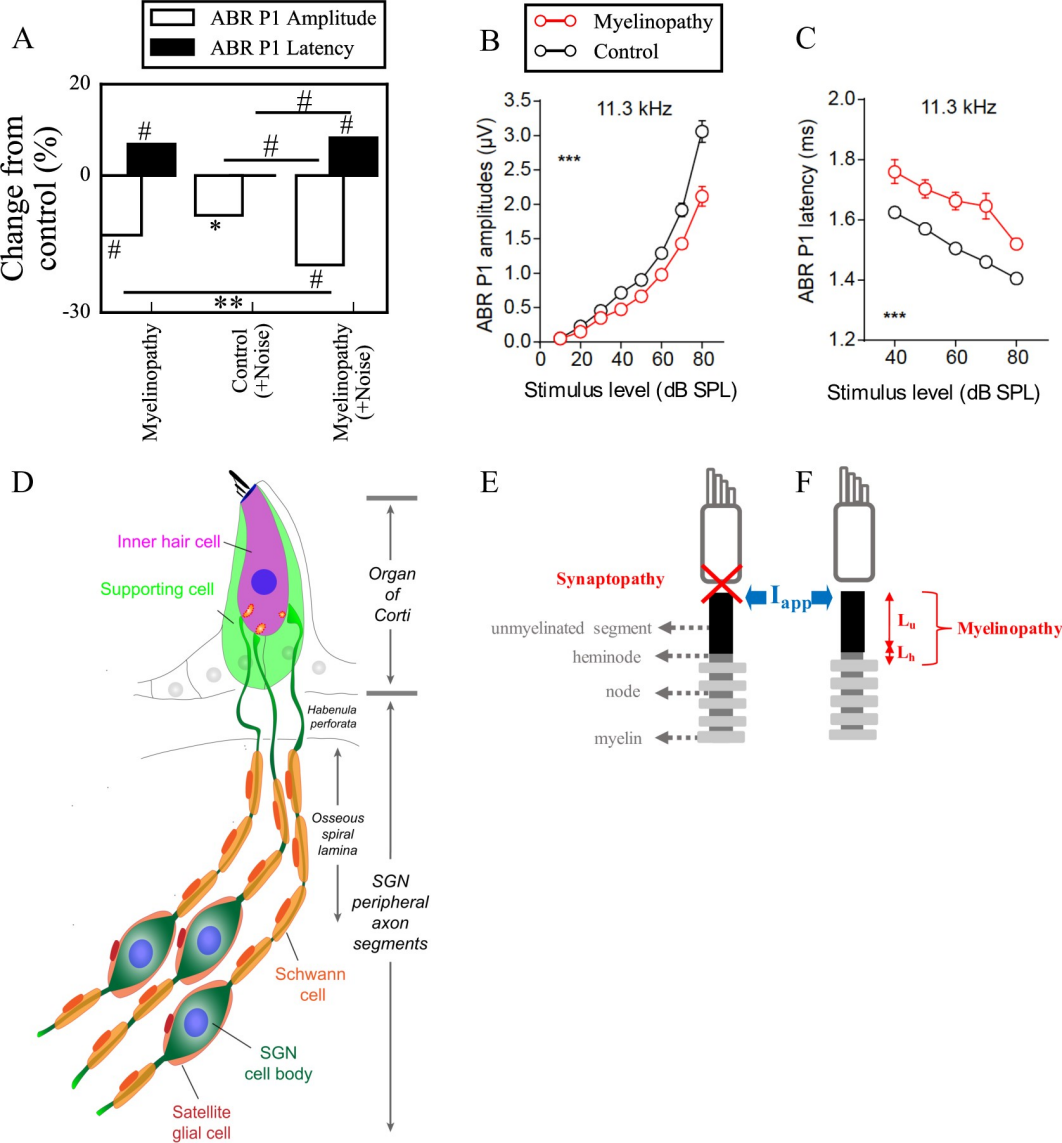
Mechanisms of hidden hearing loss. (A) Experimental results suggest that different mechanisms of HHL, myelinopathy (left) and noise exposure resulting in synaptopathy (middle), affects ABR peak 1 (P1) in distinct ways: Myelinopathy increases ABR P1 latency and decreases ABR P1 amplitude, while synaptopathy induced by noise exposure decreases ABR P1 amplitude only, without any change in latency. Combined myelinopathy and synaptopathy induced by noise exposure show additive effects (right, data taken from [8]; *p<0.05, **p<0.01, #p<0.001). Figures in panels (B) and (C) taken from [8] show ABR P1 measures evoked by 11.3kHz sound stimuli at various sound levels for control and myelinopathy cases (*** p < 0.001 by two-way ANOVA). The decrease in ABR P1 amplitude (B) in case of myelinopathy is more pronounced for higher sound levels, whereas ABR P1 latencies (C) are increased for all sound levels. (D) Schematic illustration of type I SGNs, bipolar neurons innervating IHCs via myelinated peripheral projections. (E, F) Simulated peripheral fibers of type I SGNs (SGN fiber) consist of an unmyelinated segment at the peripheral end adjacent to the site of IHC synapses, followed by a heminode and 5 myelin sheaths with 4 nodes between them. Two mechanisms of hidden hearing loss are simulated: (E) synaptopathy, modeled by removing IHC-AN synapses, and (F) myelinopathy, modeled by varying the lengths of the unmyelinated segment (*L*_*u*_) or the heminode (*L*_*h*_).

There is mounting evidence from animal studies that HHL can be caused by noise exposure and aging [6,7]. After exposure to moderate noise, animals have temporary shifts in auditory thresholds but permanent decreases in amplitude of ABR peak 1 (Fig 1A) [6–9]. Kujawa and Liberman (2009) showed that animals with this type of auditory pathology have a normal complement of hair cells and SGNs, but present with loss of a subset of synaptic connections between inner hair cells (IHCs) and SGNs. They also found that the degree of synapse loss correlates with the magnitude of the decrease in suprathreshold responses, supporting the idea that cochlear synaptopathy is a mechanism for noise-induced HHL [7]. Similar observations were made regarding aging in animal studies, i.e. HHL and synapse loss are the first signs of age-related hearing loss and have the same time-course [6]. Importantly, it has been suggested that moderate noise exposure and aging primarily affect synapses associated with high threshold/low spontaneous rate SGN fibers [10].

Auditory processing requires proper myelination of auditory nerves [11]. Therefore, it has been hypothesized that peripheral neuropathy resulting from myelin disorders may be another cause of HHL. A recent study by Wan and Corfas (2017) showed that transient demyelination causes HHL in mice [8]. In that study, acute demyelination resulted in decreased ABR peak 1 amplitudes and increased ABR peak 1 latencies without auditory threshold elevation or IHC-SGN synapse loss (Fig 1A–1C). Remarkably, these changes persisted even after remyelination of SGN fibers. Further investigation with immunostaining demonstrated that the organization of the heminodes, the nodal structures closest to the IHCs where action potentials are generated, were disrupted. These results suggest that the location of SGN heminodes is critical for normal auditory responses and that their disruption causes HHL.

In this study, we investigated the implications of these two HHL mechanisms, synaptopathy and myelinopathy, on sound-evoked spike generation and timing in SGNs. For this purpose, we constructed a reduced biophysical model consisting of a population of SGN fibers whose firing activity is driven by a previously developed, well accepted model for cochlear sound processing [12,13]. Using the model, we investigated how synapse loss or disruption of myelin organization affected spike generation and CAP properties. Synaptopathy and myelinopathy were implemented by removing synapses and by varying the position of SGN heminodes, respectively. Model results showed that heminode disruption caused decreased amplitude and increased latency of sound-evoked CAPs. In addition, significant elongation of the initial axon segment caused spike generation failure leading to decreased spiking probability. In contrast, synaptopathy, solely decreased probability of firing, subsequently decreasing CAP peak amplitude without affecting its latency, similar to observations in noise exposed animals. Model results reveal the disruptive effect of synaptopathy or demyelination on neural activity in peripheral auditory system that may further contribute to perceptual deficits.

## Methods

### SGN fiber model

Type I SGNs are bipolar neurons with peripheral axon segments innervating IHCs and central axon segments projecting into cochlear nucleus (Fig 1D) [14]. In this study, a compartmental model of peripheral axons of type I SGNs was constructed using the NEURON simulator (version 7.6.2, [15]) as schematized in Fig 1E and 1F. For simplicity, we refer to peripheral axons of type I SGNs as SGN fibers, throughout the paper. Each fiber consisted of an unmyelinated segment (length *L*_*u*_), a heminode (length *L*_*h*_) and 5 myelin sheaths following the heminode, separated by 4 nodes [16,17]. Each compartment had passive membrane properties described by specific capacitance (*C*_*m*_) and specific membrane resistance (*R*_*m*_). Specific cytoplasmic resistance (*R*_*a*_) between each consecutive compartment was modified to obtain the speed of the action potential as 3-5m/s [18], based on the neural conduction velocity measurements of rodent auditory nerve [19]. Sodium and potassium channels were inserted along the SGN fibers, except for the myelin sheaths, which only had passive membrane properties. The nominal conductances of both channel types at the unmyelinated segment was 15 times less than the nodes and the heminode [17], therefore action potentials were initiated first at the heminode. The parameters for channel dynamics were taken from [18], the stochastic channels in [18] were converted into deterministic ones for simplicity. This was done by multiplying channel density with the single ion channel conductance to obtain deterministic maximal conductance values (see Table 1 for all parameters). The Nernst potentials for the ions Na^+^ (*E*_*Na*_) and K^+^ (*E*_*K*_) were set to 66 and -88 mV, respectively, and the resting potential (*E*_*Rest*_) was -78 mV [20]. Simulations were done at 37°*C*. The differential equations were solved by fully implicit backward Euler method with time step 5μs and implemented in the NEURON simulation environment.

**Table 1:** Morphological, electrical and ion channel parameters of the different parts of a normal SGN fiber. Values as in [17] except for *R*_*a*_ and myelinated segment length which were modified for rodent SGN fibers.

Parameters	Unmyelinated segment	Heminode	Myelin	Node
**Length (μm)**	10	1	40 [modified]	1
**Diameter (μm)**	1.2	1.2	2.2	1.2
**g**_**Na**_ **(S/cm**^**2**^**)**	0.01208	0.1812	0	0.1812
**g**_**K**_ **(S/cm**^**2**^**)**	0.015	0.225	0	0.225
**R**_**m**_ **(ohm-cm**^**2**^**)**	1662	1662	1300000	1662
**C (μF/cm**^**2**^**)**	0.05125	0.05125	0.0012	0.05125
**R**_**a**_ **(ohm-cm)**	8291.4 [modified]			

*g*_*Na*_, maximal sodium conductance; *g*_*K*_, maximal potassium conductance; *R*_*m*_, specific membrane resistance; *C*, specific capacitance; *R*_*a*_, specific cytoplasmic resistance.

For each SGN fiber, the transmembrane potential *V*_*m*_ is a function of space *x* and time *t* and is expressed as:

$$-\frac{1}{R_{a}}\frac{\partial^{2}V_{m}(x,t)}{{\partial x}^{2}}+C_{m}\frac{\partial V_{m}(x,t)}{\partial t}+\frac{V_{m}(x,t)-E_{rest}}{R_{m}}+I_{ion}(x,t)=I_{app}(x,t)$$ (1)

where R_a_ is the specific cytoplasmic resistance, *C*_*m*_ is the specific capacitance, *R*_*m*_ is the specific membrane resistance, *E*_*rest*_ is the resting potential, *I*_*ion*_*(x*,*t)* and *I*_*app*_*(x*,*t)* are ionic and applied currents, respectively.

Ionic current (*I*_*ion*_*(x*,*t)*) consists of sodium (*I*_*Na*_*(x*,*t)*) and potassium (*I*_*K*_*(x*,*t)*) currents:

$$I_{ion}(x,t)=I_{Na}(x,t)+I_{K}(x,t)$$ (2)

where,

$$I_{Na}(x,t)=g_{Na}{\left(m(t)\right)}^{3}h(t)(V_{m}(x,t)-E_{Na})$$ (3)

and

$$I_{K}(x,t)=g_{K}{\left(n(t)\right)}^{4}(V_{m}(x,t)-E_{K})$$ (4)

Here, *m(t)*, *h(t)* and *n(t)* are gating variables, *g*_*Na*_ and *g*_*K*_ are maximal sodium and potassium conductances, respectively, and *E*_*Na*_ and *E*_*K*_ are the Nernst potentials for sodium and potassium ions, respectively. The gating variables *i* (for *i* = *m*, *n* and *h*) are expressed in terms of rate functions *α*_*i*_*(V*_*m*_*)* and *ß*_*i*_*(V*_*m*_*)*, such that [18]:

$$\frac{di}{dt}=\alpha_{i}(V_{m})(1-i)-\beta_{i}(V_{m})i\;for\;i=m,n,h$$ 5)

where,

$$\alpha_{m}(V_{m})=\frac{1.872(V_{m}+52.59)}{1-e^{\frac{-(V_{m}+52.59)}{6.06}}}$$ (6)

$$\beta_{m}(V_{m})=\frac{-3.973(V_{m}+57)}{1-e^{\frac{V_{m}+57}{9.41}}}$$ (7)

$$\alpha_{h}(V_{m})=\frac{-0.549(V_{m}+105.74)}{1-e^{\frac{V_{m}+105.74}{9.06}}}$$ (8)

$$\beta_{h}(V_{m})=\frac{22.57}{1+e^{\frac{-(V_{m}+22)}{12.5}}}$$ (9)

$$\alpha_{n}(V_{m})=\frac{0.129(V_{m}+43)}{1-e^{\frac{-(V_{m}+43)}{10}}}$$ (10)

$$\beta_{n}(V_{m})=\frac{-0.324(V_{m}+68)}{1-e^{\frac{V_{m}+68}{10}}}$$ (11)

### Peripheral auditory system model

We used a previously developed computational model [12,13,21] for the peripheral auditory system of guinea pig, that has fundamentally the same SGN response properties as a mouse model [22], to simulate IHC-SGN synaptic release probabilities in response to sound. Input to the model was a sound wave characterized by frequency and sound pressure level (SPL in dB). This model simulated the responses of various parts of the ear (from the middle ear to SGNs) to this sound wave. The model, as described in [21], is summarized next. A second-order linear bandpass Butterworth filter with cutoffs 22kHz and 12.5kHz was used to model the response of the middle ear to sound and compute the output stapes velocity. To simulate basilar membrane (BM) velocity in response to stapes movement, a dual-resonance-nonlinear filter bank model was used [12,13,21] and 21 BM channels are simulated. BM motion resulted in the displacement of the IHC cilia, which was approximated as

$$\tau_{C}\frac{du(t)}{dt}+u(t)=\tau_{C}C_{cilia}v(t),$$ (12)

where *u(t)* is the displacement of the IHC cilia, *v(t)* is the BM velocity, *C*_*cilia*_ and *τ*_*C*_ represent the gain factor and the time constant for the cilia displacement, respectively. The IHC cilia displacement changed the fraction of open ion channels at the IHC apical membrane, resulting in apical conductance change, which was modelled as a three-state Boltzmann distribution as

$$G(u)=G_{cilia}^{max}{\left[1+exp(-\frac{u(t)-u_{0}}{s_{0}})\times [1+exp(-\frac{u(t)-u_{1}}{s_{1}})]\right]}^{-1}+G_{a},$$ (13)

where *G(u)* is the apical conductance, $G_{cilia}^{max}$ is the maximum apical conductance and *G*_*a*_ is the passive conductance of the apical membrane. *u*_*0*_, *s*_*0*_, *u*_*1*_ and *s*_*1*_ are constants to model the nonlinearity of the proportion of open channels [12]. The IHC membrane voltage depended on its apical conductance and was modeled as

$$C_{m}\frac{dV(t)}{dt}+G(u)(V(t)-E_{t})+G_{k}(V(t)-E_{k}^{\prime })=0,$$ (14)

where *V(t)* is the IHC potential, *C*_*m*_ is the IHC membrane capacitance, *G*_*k*_ is the passive basolateral membrane conductance and *E*_*t*_ is the endocochlear potential. $E_{k}^{\prime }$ is the reversal potential of the basal current *E*_*k*_, which was described as $E_{k}^{\prime }=E_{k}+E_{t}R_{p}/\left(R_{p}+R_{t}\right),$ where *R*_*p*_ and *R*_*t*_ are the resistances of the supporting cells.

IHC membrane depolarization opened the calcium channels near the synapse, resulting in the change of calcium current (*I*_*Ca*_), which was described as

$$I_{Ca}(t)={-G}_{Ca}^{max}m_{I_{Ca}}^{3}(t)(V(t)-E_{Ca}),$$ (15)

where $G_{Ca}^{max}$ is the maximum calcium conductance near the synapse and *E*_*Ca*_ is the reversal potential of calcium. $m_{I_{Ca}}$ is the fraction of the open calcium channels, which depend on the IHC potential, given by

$$\tau_{I_{Ca}}\frac{dm_{I_{Ca}}(t)}{dt}+m_{I_{Ca}}(t)=m_{I_{Ca},\infty }$$ (16)

where

$$m_{I_{Ca},\infty }={\left[1+\beta_{Ca}^{-1}exp(-\gamma_{Ca}V(t))\right]}^{-1}.$$ (17)

Here, $\tau_{I_{Ca}}$ is calcium current time constant, $m_{I_{Ca},\infty }$ is the steady state fraction of the open calcium channels, *ß*_*Ca*_ and *γ*_*Ca*_ are constants to model experimental calcium current properties.

Calcium current (*I*_*Ca*_) changed the calcium ion concentration ([*Ca*^*2+*^]) near the synapse of the IHCs, which was modeled as

$$\frac{d[{Ca}^{2+}](t)}{dt}=I_{Ca}(t)-\left[{Ca}^{2+}](t\right)/\tau_{\left[Ca\right]},$$ (18)

where *τ*_[*Ca*]_ is calcium concentration time constant. Since calcium ions near the synapse trigger neurotransmitter release from IHCs, calcium concentration ([*Ca*^*2+*^]) affected the transmitter release rate, *k(t)*, such that:

$$k(t)=\max\left(({\left[{Ca}^{2+}\right]}^{3}(t)-{\left[{Ca}^{2+}\right]}_{thr}^{3})z,0\right),$$ (19)

where [*Ca*^*2+*^]_*thr*_ is the minimum calcium concentration required for a release and *z* is a constant to obtain release rates from calcium concentration.

The model for the IHC synapse consisted of three pools of vesicles: a cleft pool (*c*), an immediate store (*q*) and a reprocessing store (*w*). The number of vesicles in each pool was described as

$$\frac{dq(t)}{dt}=N(w(t),x)+N([M-q(t)],y)-N(q(t),k(t)),$$ (20)

$$\frac{dc(t)}{dt}=N(q(t),k(t))-lc(t)-rc(t),$$ (21)

$$\frac{dw(t)}{dt}=rc(t)-N(w(t),x).$$ (22)

These equations suggest that vesicle release occurs from the immediate store (*q*) to the cleft (*c*) at a rate *k(t)*. The vesicles in the cleft are either lost from the synapse at a rate *l*, or IHCs can take them back to the reprocessing (*w*) store at a rate *r*, where the neurotransmitters are repackaged into vesicles to be released. Then, the repackaged vesicles are transferred to the immediate store at a rate *x*. *M* in Eq 20 represents the maximum number of vesicles that can be contained at the immediate store, which receives new vesicles at a rate *y*[*M-q(t)*]. Here, the rate of the transfer of vesicles from the reprocessing store to the immediate store (*x*) and the rate that the immediate store receives new vesicles (*y*) are constants taken from [13,21].

**Fig 2:**
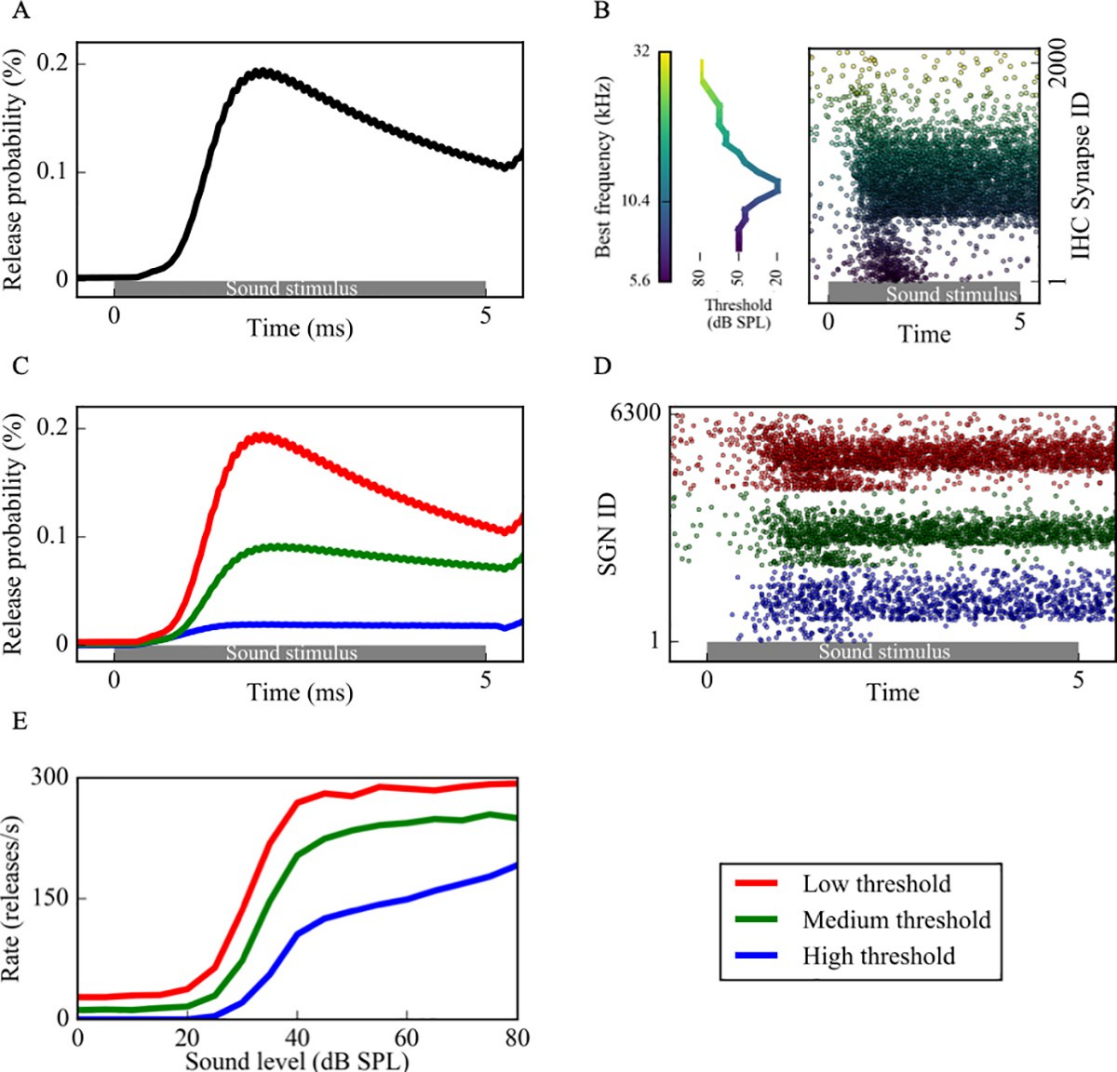
Sound-evoked activity of low, medium and high threshold SGN fibers resulting from increased vesicle release probabilities from corresponding IHC-SGN synapses. (A) Sound stimuli increased vesicle release probability from IHCs (as computed using the coupled (Eqs 12–22)) and release times were determined by a Poisson process. (B) Cumulative release events of an IHC synapse population with best frequencies (BFs) between 5.6kHz-32kHz in response to a 10kHz sound stimulus (right). The dots, color coded based on the BFs of the synapses, represent release times at each IHC synapse in a population of 2000. Since the thresholds of IHCs depend on their BFs (left), each synapse has a different release pattern. For each release event, the associated SGN fiber is stimulated with a brief external current pulse, resulting in spiking activity. (C) Three groups of SGN fibers, low (LT), medium (MT) and high (HT) threshold, were simulated based on their spontaneous firing rates and saturation profiles in response to sound. (D) Based on the release probabilities, different fiber types exhibit different cumulative responses (red dots: low threshold, green dots: medium threshold, blue dots: high threshold). Panels A-D are example simulations for simulated 80dB SPL 10kHz sound stimuli. (E) The increase in spike rates of simulated fiber type in response to increasing sound levels is comparable to Fig 3 of [12].

In this IHC synapse model, vesicles in the cleft and the reprocessing store are continuous quantities, whereas the immediate store has quantal vesicles, whose release is a stochastic process. This process is described by the function *N(n*, *ρ)*, which means that there are *n* vesicles, each having a release probability of *ρdt* at a single time step *dt*. As a result, we take the term *N(q(t)*, *k(t))* as an output of this model, which gives us the release rate, i.e. the release probability from IHCs. In our model, we used the excitation protocol from [8] and applied pure tone, 10kHz 5 ms long sound stimuli. As a result, we simulated the stimulus mediated release probabilities expected from that excitation (Fig 2A). We used the same parameters as in [13,21], except for those listed in Table 2, in order to obtain release rates similar to the experimental results as in Fig 3 in [12]. Since release events into the IHC-SGN synapses are mediated by calcium currents in IHCs in the vicinity of the IHC synapse [23,24], we varied maximum calcium conductance near the IHC synapse ($G_{Ca}^{max}$) to define different fiber types.

**Table 2:** Parameters for the model of middle ear-IHC synapse, changed from [13,21].

Parameters	LT	MT	HT
**Number of the linear gammatone filters**	3	3	1
$G_{Ca}^{max}$ **(nS)**	4	3	2

$G_{Ca}^{max}$, maximum calcium conductance near the IHC synapse; LT, low threshold fiber; MT, medium threshold fiber; HT, high threshold fiber.

The output of the IHC synapse model was IHC-SGN synaptic release probabilities (Fig 2A), which were used to determine a Poisson process of IHC release (Fig 2B) that governed brief external stimuli to the corresponding nerve fiber to induce action potential generation. The external stimuli mimicking the post-synaptic response to synaptic release from IHCs [25] were simulated by external current pulses *I*_*app*_, of the form

$$I_{app}=0.39A{(e}^{-\frac{t}{\tau_{2}}}-e^{-\frac{t}{\tau_{1}}})(V_{m}-E_{exp})$$ (23)

where *A* = 0.12nS, *τ*_*1*_ = 0.1ms, *τ*_*2*_ = 0.3ms and E_exp_ = 0mV, unless otherwise stated (S4 and S5A Figs). *I*_*app*_ was applied at the beginning of the unmyelinated segment (Fig 1E and 1F) and the time of the action potential at the center of the heminode was taken as output (Fig 2D).

### Defining different fiber types

SGNs can be classified into 3 groups depending on their spontaneous firing properties, thresholds for sound-evoked activity and saturation profiles, namely low threshold (LT), medium threshold (MT) and high threshold (HT) fibers. Based on the measurements reported in [26], we modeled the properties of these three fiber groups as follows (Fig 2C–2E): LT fibers have high spontaneous rates (18–100 spikes/s), low dynamic ranges, and reach their maximum discharge rate within approximately 30 dB sound pressure level (SPL). MT fibers have lower spontaneous firing (between 0.5 and 18 spikes/s), higher dynamic ranges, and show slower increase and saturation of spike rates with increasing SPL compared to LT fibers. HT fibers have very low spontaneous firing rates (<0.5 spikes/s), and response thresholds higher than ~20 dB SPL, which is the highest threshold of all 3 groups. For higher SPL, their spike rate increases linearly with sound intensity, therefore their dynamic range is the highest [26]. To simulate these different fibers, we varied [*Ca*^*2+*^]_*thr*_ and $G_{Ca}^{max}$ of the IHC synapses of each fiber type (see [13,21] and Table 2). In our model, we had IHC synapses with 21 characteristic frequencies varying between 5.6kHz and 32kHz distributed according to the Greenwood map [21,27] to simulate experimental data [8]. For each characteristic frequency, we used 100 LT, 100 MT and 100 HT fibers with 6300 fibers in total. Each fiber received input from an individual synapse, whose activity is modeled as explained above.

### Analyzing simulated spike trains

Simulated sound stimulus generates a sequence of spikes in each model SGN fiber (Fig 2D). We used three methods to analyze SGN fiber spike trains:

#### Measurement of time intervals between non-identical spike trains of SGN fiber populations

**Fig 3:**
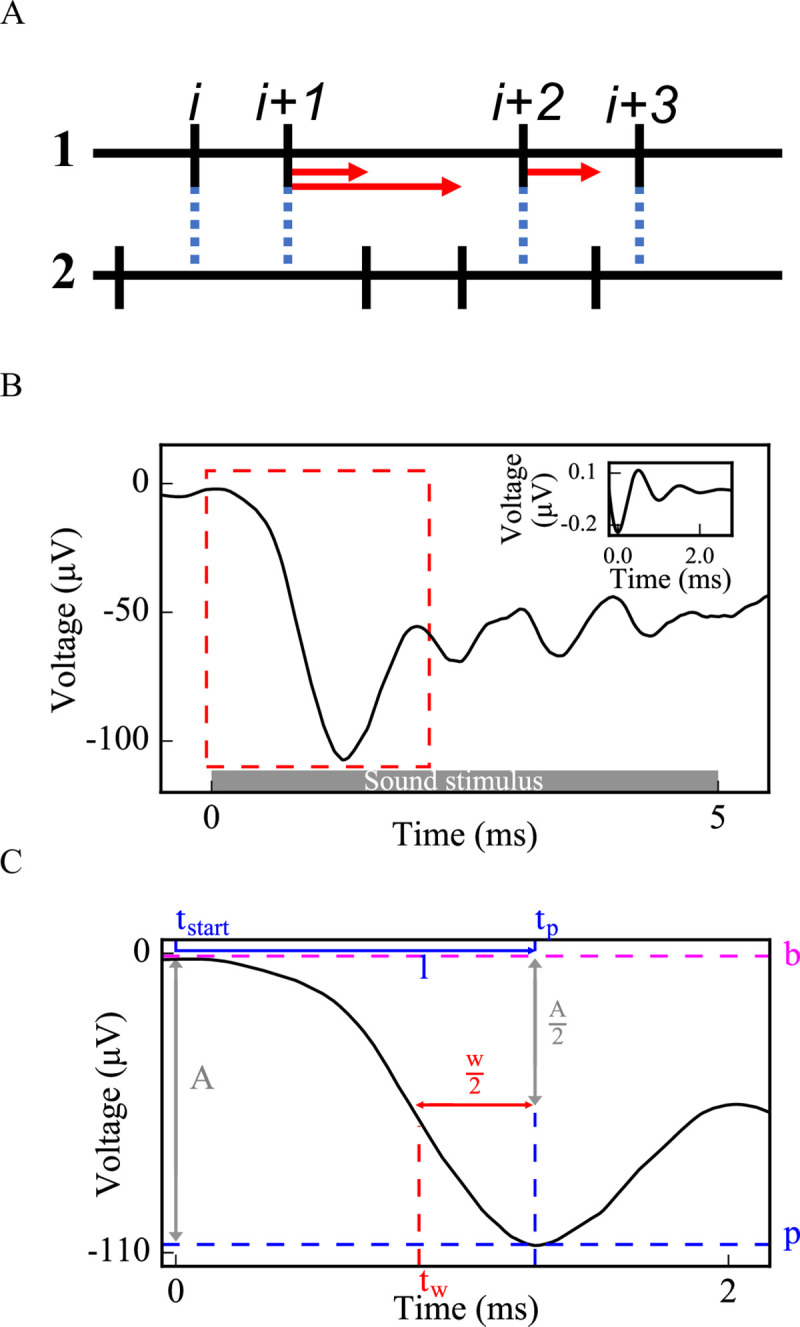
**Methods used to evaluate cumulative activity of SGN fiber populations: pairwise spike time differences (A) and simulated CAP (B,C).** (A) For each non-identical pair of spike trains (1 and 2) from an SGN fiber population, forward time intervals were measured between each spike *i* of spike train 1 and all spikes of spike train 2 falling between times of spikes *i* and *i+1*. Standard deviations of the distributions of these time intervals were calculated to evaluate synchronous spike timing in the SGN fiber population. (B) Each spike in Fig 2D was convolved with the unitary response of a CAP [the inset of (B)] and convolutions from each spike were summed up to obtain a simulated CAP of the SGN fiber population. (C) Amplitude, latency and width were measured from the first peak of the simulated CAP [dashed rectangle in (B) is zoomed in for (C)] (*b*: baseline, *p*: peak, *A*: amplitude of the peak, *t*_*p*_: peak time, *l*: latency, *w*: width, *t*_*w*_: half amplitude time before *t*_*p*_).

This metric, modified from a shuffled autocorrelogram measure in [28], was used to quantify temporal properties of SGN fiber spiking within a population based on the time intervals of the spikes between each non-identical pair of spike trains within the population. From all possible non-identical pairs of spike trains within a population, forward time intervals were measured between each spike *i* of the first spike train and spikes of the second spike train falling between the *i-*th and (*i+1)*-st spikes (Fig 3A). All time intervals from all pairs were tallied in a histogram. The histogram was reflected about the y-axis, since each forward time interval of a pair (a,b) is a backward time interval of the pair (b,a).

#### Convolution into the unitary response of compound action potential (CAP)

To yield a cumulative response of the activity of the population of SGN fibers and to be able to compare model results with in vivo ABR peak 1 (P1) results, we convolved each spike with the unitary response and summed them up to generate a population CAP (Fig 3B). In this study, we considered this computed CAP as equivalent to ABR P1. The unitary response *U(t)* was described as in [29]:

$$U(t)=\left\{ \begin{array}{c} A\times e^{-k(t-0.288)}\times \sin(2\pi f(t-0.288))\\ 0 \end{array}\right.$$

for−0.215≤t≤2.785 (24)

otherwise

where *A = 0*.*16μV*, *k = 1*.*44ms*^*-1*^, *f = 0*.*994ms*^*-1*^ and *t* is the time (Fig 3B inset).

Fifty population CAPs were averaged to measure the width (*w*), amplitude (*a*) and latency (*l*) of the initial CAP peak more accurately, which were computed as:

a=|p−b| (25)

$$l=t_{p}-t_{start}$$ (26)

$$w={2(t}_{w}-t_{p})$$ (27)

where *p* is the peak voltage, *b* is the baseline voltage, *t*_*p*_ is the time when the voltage equals *p*, and *t*_*w*_ is the time when the voltage equals $-\left(|b|+\frac{a}{2}\right)$ (the half-peak) before *t*_*p*_ (Fig 3C).

#### Calculating spike probability and latency for each SGN fiber population

The probability that release events at IHC-SGN synapses resulted in spikes at the heminodes of an SGN fiber population was calculated by dividing the number of spikes at the heminode of each SGN fiber by the number of release events and averaging over all fibers within a population. Spike latency of an SGN fiber population was calculated by the time difference between a spike and a release preceding that spike averaged over all spikes of that population.

## Results

Using the model of the type I SGN fiber population, we investigated the effects of myelinopathy and synaptopathy on type I SGN spike generation and spike timing. We first simulated different myelinopathy scenarios by varying the length of the initial unmyelinated segment *L*_*u*_ (Fig 1F, from a putative control value of 10 μm) and the first heminode length *L*_*h*_ (from a control value of 1 μm) for all (i.e. LT, MT and HT) fibers. Next, we simulated synaptopathy by removing IHC-SGN synapses (Fig 1E) considering the cases where only synapses on HT fibers were affected or synapses on all fiber types were affected. Lastly, we investigated the combined effects of myelinopathy and synaptopathy.

### Effects of myelinopathy on SGN population activation patterns

**Fig 4:**
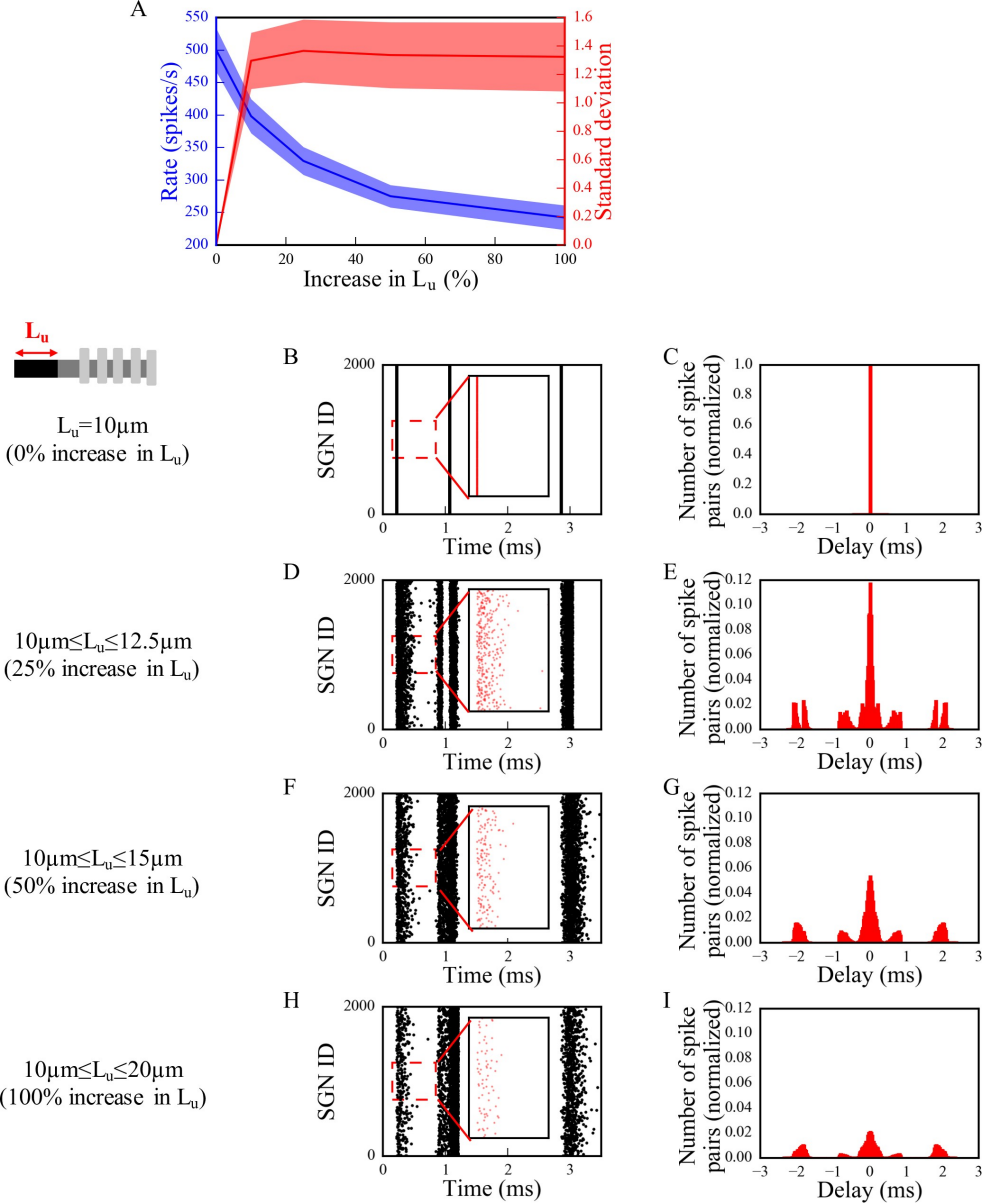
The synchronous activity of SGN fiber populations is disrupted and their response to sound is decreased with increasing levels of *L*_*u*_ heterogeneity. SGN fiber populations with different heterogeneity levels of *L*_*u*_ were stimulated with 80dB sound stimulus at 0ms. We assumed release events from all IHCs for the population occurred simultaneously. Firing rate and standard deviations of time intervals are averaged for all populations in (A), shaded area represents the standard error of the mean. Raster plots [(B), (D), (F) and (H), insets: the first bursts of the raster plots] and corresponding histograms of time intervals between non-identical pairs of spike trains within a population normalized to the total number of spike pairs in panel B [(C), (E), (G) and (I)] are shown for populations of SGN fibers with *L*_*u*_ = 10μm (0% increase in *L*_*u*_) [(B) and (C)], 10μm≤*L*_*u*_≤12.5μm (25% increase in *L*_*u*_) [(D) and (E)], 10μm≤*L*_*u*_≤15μm (50% increase in *L*_*u*_) [(F) and (G)] and 10μm≤ *L*_*u*_≤20μm (100% increase in *L*_*u*_) [(H) and (I)]. The ordinates of the histograms are normalized over the number of spike pairs with 0ms delay for the population where all fibers have *L*_*u*_ = 10μm (C). Simulations were done 10 times.

Mouse studies have shown that transient demyelination and the subsequent remyelination alters the position of SGN heminodes, resulting in heminodes that are positioned farther from the IHC-SGN synapse and at variable positions, in contrast to healthy SGN fibers where heminodes on all fibers are aligned [8]. To identify the effect of this heterogeneity of heminode locations on SGN spike timing, we first considered a population of fibers with different ranges of *L*_*u*_ values stimulated with identical IHC release patterns (Fig 4). Here, we denote 0% increase as the putative control fiber length (*L*_*u*_ = 10 μm), while 100% increase means *L*_*u*_ was varied between 10 and 20 μm uniformly across the population. We assessed the level of synchronization of spikes across the LT SGN fiber population with ~10kHz characteristic frequency by stimulating all fibers with an identical IHC release pattern in response to 80dB SPL sound stimulus. This means, each fiber received the same synaptic input. We stimulated all fibers at time 0ms (Fig 4) with the excitation protocol from [8], as explained in Methods. As heterogeneity of *L*_*u*_ values was increased (Fig 4A), the population spike rate decreased reflecting spike generation failure on fibers with large *L*_*u*_. At the same time, variability in spike timing increased as illustrated in spike raster plots (Fig 4B, 4D, 4F and 4H show a portion of the generated spike trains, insets show timing of first spikes) and computed pairwise spike time intervals (Fig 4C, 4E, 4G and 4I; see Methods). These disruptions in spike generation and timing resulted in increased standard deviation of the distribution of pairwise spike time differences across the population (Fig 4A). These initial observations suggest that myelinopathy not only disrupts spike timing of SGNs within a population, but also leads to the loss of spikes.

**Fig 5:**
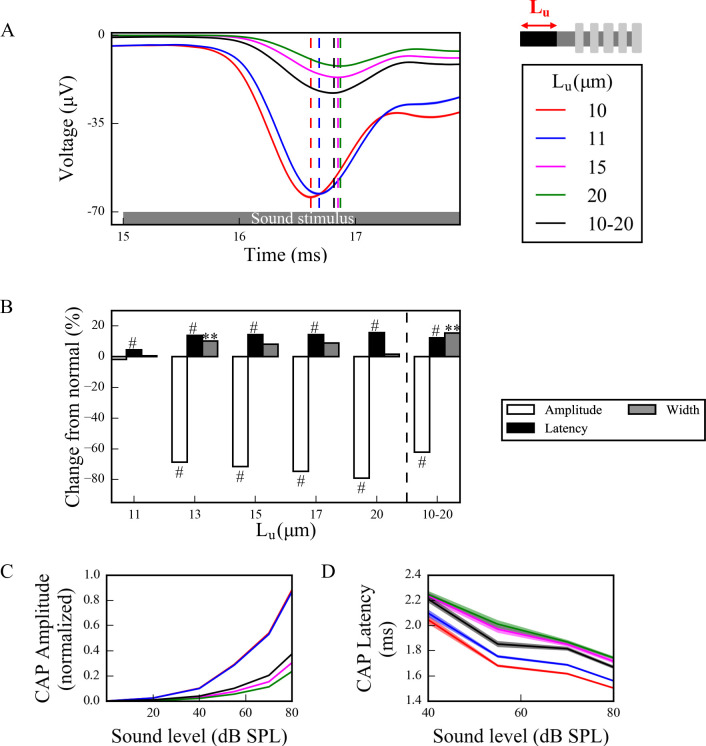
Longer *L*_*u*_ significantly decreases and delays the peak of the sound-evoked CAPs of SGN fibers. (A) Sound-evoked CAPs of SGN fiber populations with varying unmyelinated segment length *L*_*u*_ at 70dB SPL, averaged over 50 simulations. Shaded regions correspond to the standard error of the mean and dashed lines correspond to the peaks of each CAP, labeled with the same colors as the CAPs. The decrease and delay of peak CAPs were significant for populations with *L*_*u*_ > 11 μm. (B) Comparison of CAP measures of each population relative to normal *L*_*u*_ (*L*_*u*_ = 10 μm) at 70 dB SPL. Latencies were significantly higher for populations with *L*_*u*_>10 μm and peaks were significantly lower for populations with *L*_*u*_>11 μm. The increases in widths were only minimal, however significant for the heterogeneous population, where 10 μm ≤ *L*_*u*_ ≤ 20 μm (*p<0.05, **p<0.01, #p<0.001). (C) Normalized CAP amplitudes for various sound levels exhibited an exponential increase and the decreases in CAP amplitudes for populations with *L*_*u*_>11 μm were more pronounced for higher sound levels. (D) The latencies of CAP peaks increased with higher *L*_*u*_ for all sound levels and decreased along the sound levels.

**Fig 6:**
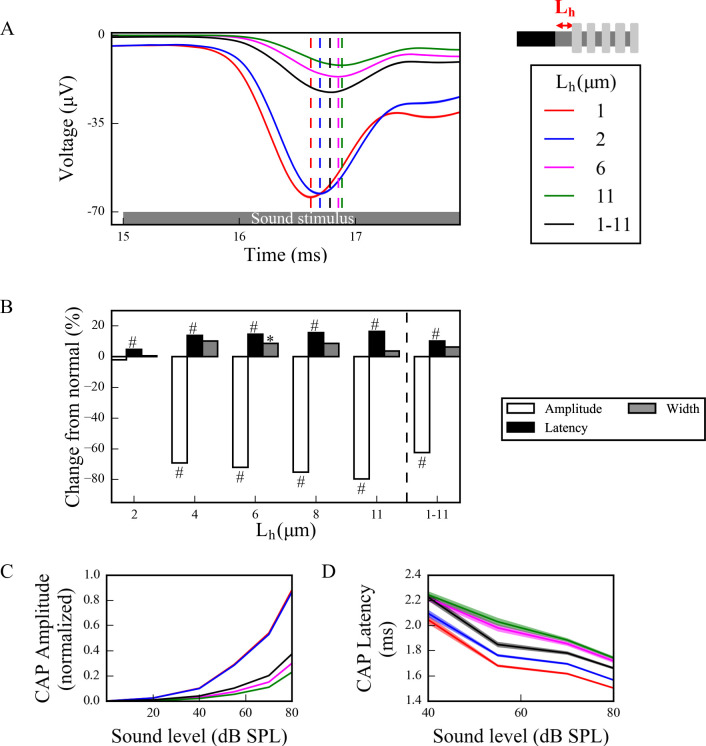
Longer *L*_*h*_ significantly decreases and delays the peak of the sound-evoked CAPs of SGN fibers. (A) Sound-evoked CAPs of SGN fiber populations of varying heminode length *L*_*h*_ at 70dB SPL, averaged over 50 simulations. Shaded regions correspond to the standard error of the mean and dashed lines correspond to the peaks of each CAP, labeled with the same colors as the CAPs. The decreased peak amplitude and increased latency of CAP peak were significant for populations with *L*_*h*_ > 2 μm. (B) Comparison of CAP measures of each population relative to the normal *L*_*h*_ (*L*_*h*_ = 1 μm) at 70 dB SPL. CAP latencies were significantly higher for populations with *L*_*h*_>1 μm and peak amplitudes were significantly lower for populations with *L*_*h*_>2 μm. The increases in widths were only minimal (*p<0.05, **p<0.01, #p<0.001). (C) Normalized CAP amplitudes exhibited an exponential increase and the decreases in CAP amplitudes of populations with *L*_*h*_>2 μm were more pronounced for higher sound levels. (D) The latencies of CAP peaks increased with higher *L*_*h*_ for all sound levels and decreased along the sound levels.

To investigate effects of this disruption of spike generation and timing in the full model, CAPs were computed from spike responses of populations of LT, MT and HT SGN fibers subject to simulated myelinopathy. Responses of fiber populations with homogeneous initial unmyelinated segment length (*L*_*u*_) or first heminode length (*L*_*h*_) values were investigated to see the gradual effect of variable myelination patterns on cumulative activity of SGN fibers. Additionally, populations with heterogeneous, random *L*_*u*_ or *L*_*h*_ values were simulated to represent a population heterogeneity induced by myelinopathy. We note that when increasing first heminode length (*L*_*h*_) the number of expressed channels (Na^+^ and K^+^) was kept constant, consequently decreasing their density. However, when increasing initial unmyelinated segment length (*L*_*u*_), the density of expressed channels was kept constant, consequently increasing their number. Results were not qualitatively different when these assumptions were reversed (see Discussion section). Model results show that, in response to a simulated 70 dB SPL stimulus, CAPs computed from SGN fiber populations with homogeneous myelination patterns had decreased peak amplitude and increased latency to the peak when *L*_*u*_ was longer than the putative normal length of 10 μm (Fig 5A) and *L*_*h*_ was longer than the putative normal length of 1 μm (Fig 6A). The latency of the simulated CAP for a normal SGN population (*L*_*u*_ = 10 μm and *L*_*h*_ = 1 μm) was ~1.5ms, which is within the range of experimental CAP latencies [29]. The amplitude decrease was highly significant for *L*_*u*_ > 11 μm and *L*_*h*_ > 2 μm with ~70% of a drop from normal (Figs 5B and 6B). This was due to the fact that at those values failure of spike generation occurred because of the increased lengths, *L*_*u*_ and *L*_*h*_. CAP peak latencies were significantly longer than normal for all homogeneous populations, with *L*_*u*_ > 11 μm and *L*_*h*_ > 2 μm having ~20% of an increase. The changes in CAP widths were minimal for all cases. For populations with heterogeneous myelination patterns, however, CAP peaks were significantly (~60%) lower, and latencies and widths were significantly higher than normal populations (Figs 5B and 6B).

In addition, to assess the dependencies of CAP properties on sound intensities, we measured responses to simulated sound stimuli between 0–80 dB. For *L*_*u*_ ≤ 11 μm and *L*_*h*_ ≤ 2 μm, CAP peak amplitudes increased with sound intensity (Figs 5C and 6C, respectively) and the profile of increase was more similar to experimental measurements of ABR (Fig 1B, also see S4 Fig in [8]) and CAP [29]. However, for *L*_*u*_ > 11 μm and *L*_*h*_ > 2 μm, CAP amplitudes remained small for all sound intensities due to reduced spike generation. For populations with heterogeneous myelination patterns, CAP amplitudes were between the *L*_*u*_ = 11 μm and *L*_*u*_ = 15 μm cases, and the *L*_*h*_ = 2 μm and *L*_*h*_ = 6 μm cases for all sound levels, reflecting reduced spike generation in some fibers of the population with higher *L*_*u*_ and *L*_*h*_ values. CAP latencies were longer for higher values of *L*_*u*_ and *L*_*h*_ (Figs 5D and 6D) and they decreased with increasing sound levels for all cases, consistent with experimental observations (Fig 1C, also see S4 Fig in [8]). In the heterogeneous populations, CAP latencies showed values between the *L*_*u*_ = 11 μm and *L*_*u*_ = 15 μm cases, and the *L*_*h*_ = 2 μm and *L*_*h*_ = 6 μm cases.

### Effects of synaptopathy on SGN population activation patterns

**Fig 7:**
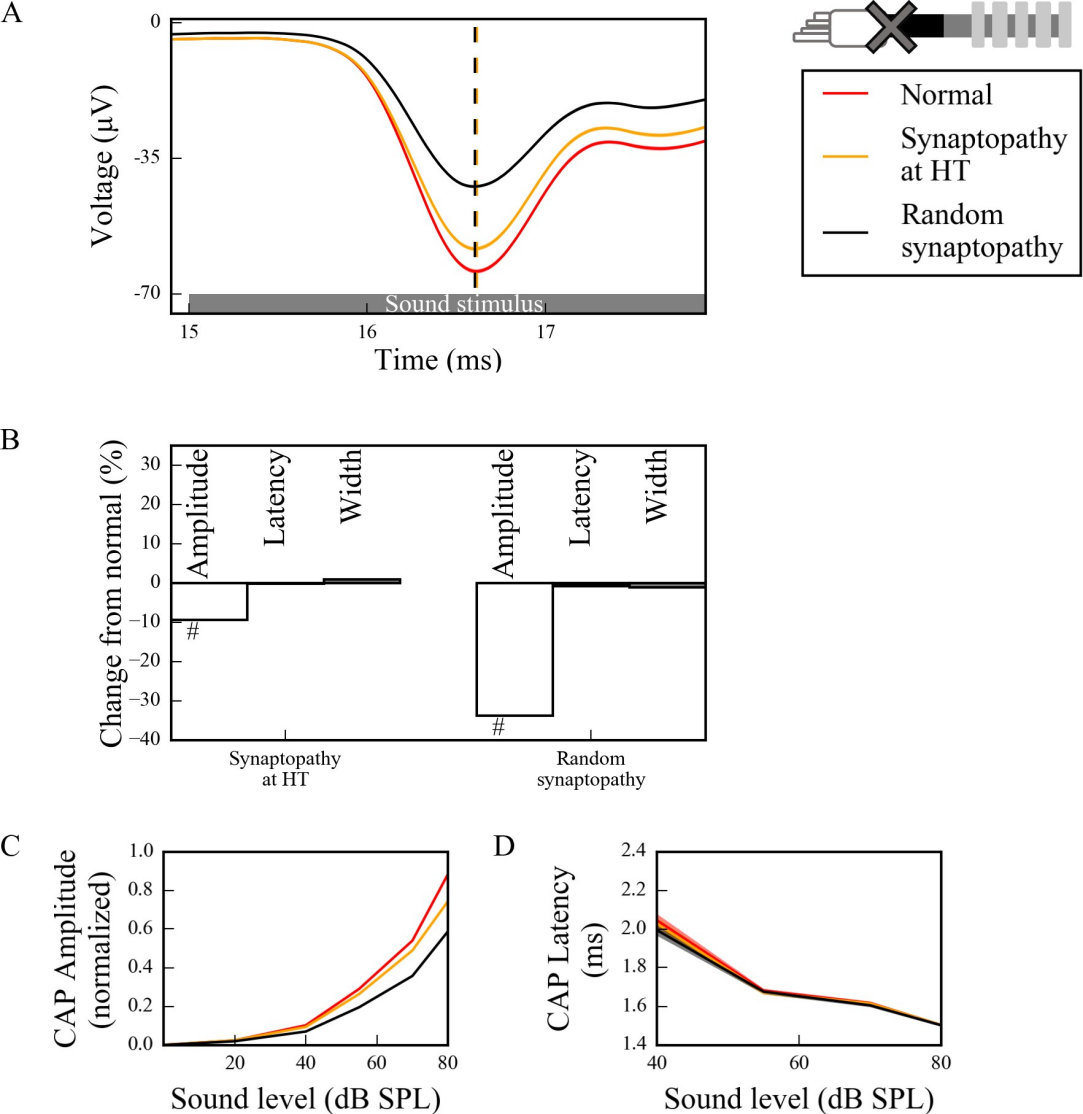
Synaptopathy at IHC-SGN synapses decreases the peak of the CAP significantly, without changes to peak latency and width. (A) Sound-evoked CAPs of SGN fiber populations with different synaptopathy scenarios at 70dB SPL, averaged over 50 simulations. Shaded regions correspond to the standard error of the mean and dashed lines correspond to the peaks of each CAP, labeled with the same colors as the CAPs. Synaptopathy had smaller effects on CAP peak amplitude and latency when it affected only HT fiber synapses compared to affecting all fiber types randomly. (B) Comparison of CAP measures of synaptopathy cases relative to normal (no synaptopathy) at 70 dB SPL (*p<0.05, **p<0.01, #p<0.001). (C) Normalized CAP amplitudes exhibited an exponential increase and the decreases in CAP amplitudes of populations with both synaptopathy scenarios were more pronounced for higher sound levels. The latencies of the CAP peaks did not exhibit any significant difference between different populations for all sound levels.

There is strong evidence indicating that noise-induced synaptopathy, primarily at HT fibers, could be one of the mechanisms of hidden hearing loss [10,30,31]. To simulate it, we considered responses of a population of control SGN fibers (*L*_*u*_ = 10 μm, *L*_*h*_ = 1 μm) with all HT IHC-SGN synapses removed. To investigate the specific effect of loss of synapses on HT fibers, we compared responses to the case where the same number of synapses (1/3^rd^ of whole population) were removed randomly from the whole population of three fiber types. The CAPs computed from populations with and without synaptopathy (Fig 7A) in response to a 70 dB SPL suggest that HT-targeted synaptopathy produces only a small effect on CAP peak amplitude while random synaptopathy has a more significant effect on the amplitude (~30% vs ~10% decrease from normal at 70dB SPL) (Fig 7B). Moreover, there were no latency or width changes for both HT and random synaptopathies.

We simulated sound intensities between 0–80 dB SPL to assess how CAP peak amplitude and latency depended on sound intensities in the simulated synaptopathy model. For HT synaptopathy, a decrease of CAP peaks was observed for only higher sound intensities (>70dB SPL), while CAP peaks for random synaptopathy were lower than the normal case for sound intensities higher than 40dB SPL (Fig 7C). CAP latencies did not show any significant differences for any sound level in any synaptopathy case (Fig 7D).

### Combined effects of myelinopathy and synaptopathy of hidden hearing loss

**Fig 8:**
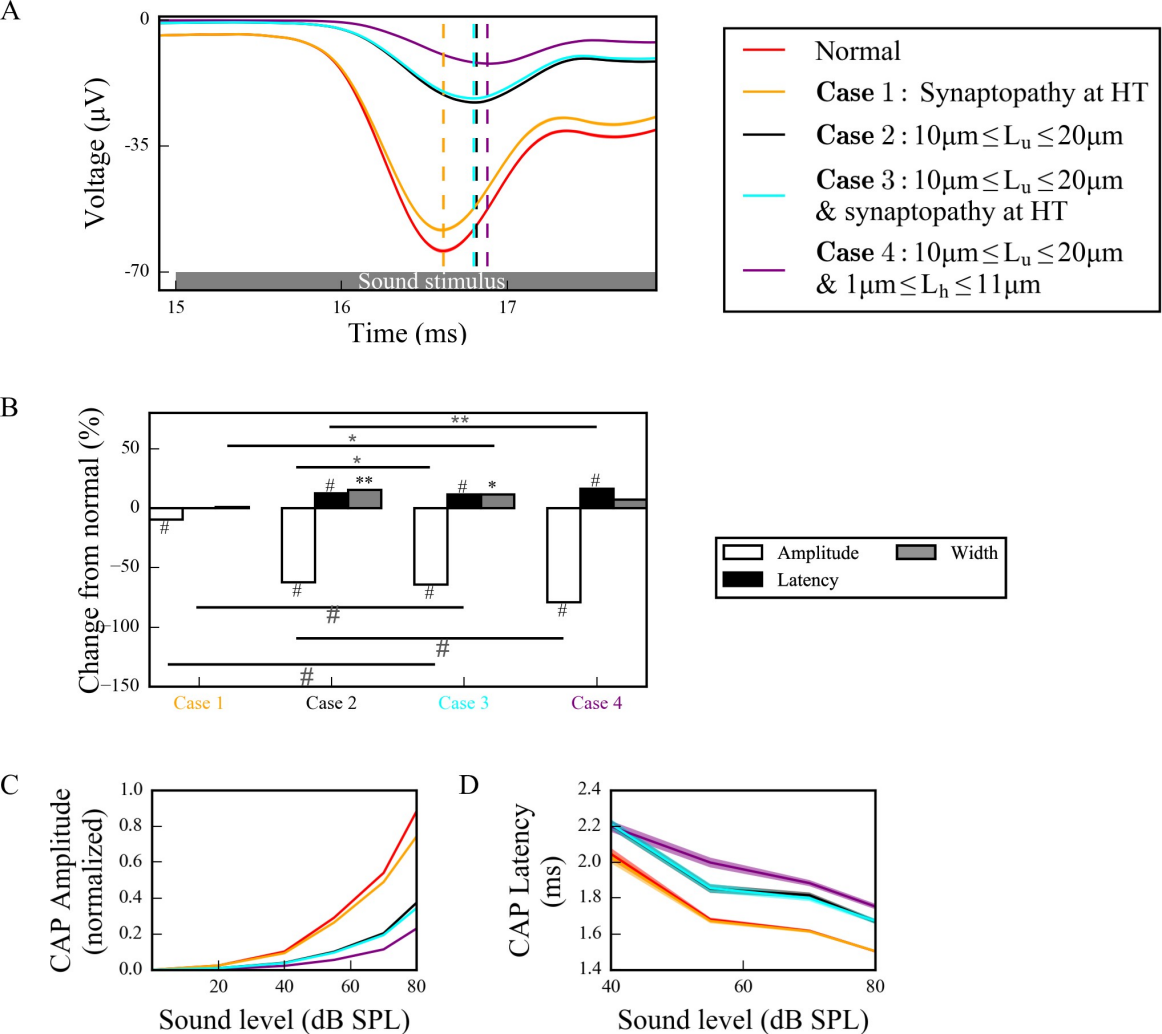
Different scenarios of hidden hearing loss have additive effects on SGN activity. (A) Sound-evoked CAPs of SGN fiber populations with different myelinopathy and synaptopathy scenarios at 70dB SPL, averaged over 50 simulations (dashed lines correspond to the peaks of each CAP, labeled with the same colors as the CAPs). Combined synaptopathy and myelinopathy (Case 3) showed additive effects on the decrease in CAP peak amplitude, but not on the increase in CAP peak latency (compare to Cases 1 and 2). Combined different myelinopathies showed additive effects on both CAP peak amplitude and latency (compare Cases 2 and 4). (B) Comparison of average CAP measures for different myelinopathy and synaptopathy cases relative to normal, and between cases at 70 dB SPL (*p<0.05, **p<0.01, #p<0.001). Normalized CAP amplitudes (C) and CAP latencies (D) for different myelinopathy and synaptopathy cases for various sound levels, averaged over 50 simulations. Shaded areas correspond to the standard error of the mean.

To investigate how different proposed HHL mechanisms interact and affect cumulative SGN fiber activity, we combined them in our model (Fig 8). When HT synaptopathy (Fig 8A and 8B) was combined with myelinopathy affecting the length of the initial unmyelinated segment *L*_*u*_, CAP peak amplitude showed significant additive decrease, but latency and width showed no change beyond that produced by the myelin defects alone (compare Case 3 with Cases 1 and 2). When both myelinopathy mechanisms were combined by varying *L*_*u*_ and *L*_*h*_ across the population, both CAP peak amplitude and latency showed significant additive changes (compare Case 4 with Case 2). At 70dB SPL simulated sound stimulus, CAP widths were significantly increased only by myelinopathy mechanisms. In response to varied sound intensities between 0–80 dB SPL, the additive effects of synaptopathy and myelinopathy on CAP peak amplitude and latency changes were prominent for higher SPL (Fig 8C and 8D, respectively).

In summary, model results suggest that decreases in CAP peak amplitudes show additive effects for combined synaptopathy and myelinopathy. Also, there were significant increases in CAP peak latencies and CAP widths only for myelinopathy-based mechanisms, with latencies showing additive effects in combined myelinopathies, while synaptopathies do not affect this CAP feature.

## Discussion

We built a reduced biophysical model simulating sound-evoked activity of type I SGN populations to analyze two hypotheses for the cause of HHL, synaptopathy and myelinopathy. Inner hair cell synaptic release in response to sound was simulated using the well-accepted model developed in [12,13] and induced firing of modeled SGN axons. SGN spike times were convolved with the unitary response of the CAP, a near-field response of SGNs, to convert spike times into cumulative activity for comparison with experimental results. The cumulative CAP for the normal case (*L*_*u*_ = 10 μm and *L*_*h*_ = 1 μm) in our model had similar characteristics as experimentally measured CAPs [29]: The peak latency at 80dB SPL was ~1.5ms and the peak amplitude increased exponentially with increasing sound levels, with a value of ~70μV at 70dB SPL. Simulating each mechanism for HHL led to similar changes in CAP responses as reported experimentally. Specifically, model results showed that synaptopathy reduced the amplitude of the cumulative CAP response without affecting its latency, as reported in [1,7], due to a reduction in the number of responsive nerve fibers without disruption of spike timing. In contrast, myelinopathy, when modeled as disorganization of either the initial unmyelinated nerve segment length or the heminodal spacing, caused disruption of spike timing in addition to loss of firing response, affecting both the peak amplitude and latency of the cumulative CAP, as reported in [8].

In this study, we focused on the definition of HHL used in animal models, namely the observation that both synaptopathy and myelinopathy lead to reduced amplitude of the CAP (i.e. peak 1 of the ABR response) without ABR threshold shifts. In animal models, the relationship between synaptopathy or myelinopathy and perceptual defects has not yet been explored. In contrast, studies of HHL in humans focus primarily on the relationship between normal audiometry thresholds and deficits in complex sound or speech perception in noisy environments. Evidence in human studies of noise-exposed presumptive AN damage resulting in ABR and perceptual deficits despite normal auditory thresholds has been mixed with some studies showing effects [4,5] and some not [2,3]. On the other hand, individuals with peripheral neuropathies that affect myelination, such as Guillain-Barré Syndrome (GBS) [32] and Charcot-Marie-Tooth (CMT) disease [33], have been reported to have perceptual difficulties even when having normal auditory thresholds, suggesting HHL. Nevertheless, given the robust physiological and anatomical data for HHL generated in animal studies, our mathematical model focuses on effects on CAPs and not on perception.

As shown by Wan and Corfas (2017), myelinopathy affects the distance from the IHC-SGN synapse to the heminode and introduces heterogeneity in heminode locations across a SGN fiber population [8]. Here, we provided evidence that increasing heterogeneity of heminode locations decreases the synchronization of spike timing of SGN fiber populations. Moreover, spike rates of more heterogeneous SGN fiber populations dropped, due to a loss of spike generation in SGN fibers with heminodes further from IHCs (Fig 4). Our simulations of cumulative CAP signals showed that the amplitude of the simulated CAP decreased with myelinopathy, reflecting the reduction of SGN spike activity. In addition, myelinopathy increased the latency and the width of the CAP peak, similar to experimental observations of ABR P1 (Fig 1C), providing support for the disruption of spike timing in SGN activity (Figs 5 and 6). However, it should be noted that increased latency of the peak of CAP may also result from outer hair cell (OHC) deficits [34,35]. Therefore, a CAP latency could be diagnosed as myelinopathy as long as OHCs are normal, which is the case in our simulations and the experimental data we referenced [8].

Previously, it has been shown that noise exposure and aging caused HHL due to synapse loss at SGN-IHC synapses, which resulted in a decrease of ABR P1 without increases in latency or thresholds [6–8,36]. Moreover, it has been hypothesized that synapse loss occurs preferentially at HT SGN-IHC synapses [10]. Consistent with experimental results, our simulations for both HT synaptopathy and random synaptopathy showed that CAP latencies were unchanged for either scenario, but the amplitude of the CAP peak was significantly decreased. These results show that the effect of either synaptopathy scenario on CAP properties is consistent with experimental observations (Fig 7) [6–8].

A computational study from Bourien et al. (2014) previously investigated the effects of different auditory fiber degeneration scenarios on cumulative CAP characteristics [29]. They provided evidence that removing 1/3^rd^ of the auditory fibers randomly from an auditory fiber population linearly decreased the cumulative CAP amplitude with minimal threshold elevation and no change in CAP peak latency, which is consistent with our results for random synaptopathy (Fig 7). They also removed preferentially HT fibers from the population and showed that there was no threshold elevation and no change in CAP peak latency and amplitude, when all HT fibers were removed. This differs slightly from our results for HT synaptopathy, since we saw a decrease in CAP peak amplitude when we removed all HT synapses (Fig 7). This might be due to the difference in HT fiber proportions in both studies; in our case 1/3^rd^ of the synapses were HT, whereas in their study only 10% were HT.

Combining synaptopathy and myelinopathy HHL mechanisms led to additive effects in our model. Decreases in CAP peak amplitude were additive for combined synaptopathy and myelinopathy, but synaptopathy did not contribute to changes in CAP latency even in the combined scenario. Combining myelinopathy mechanisms led to additive increases in both peak CAP amplitude and latency (Fig 8). These results match qualitatively with the experimental results (Fig 1A), further supporting the accuracy of our model. Further, we did the same simulations with modified rate-response curves for different fiber types (S1A Fig) so that the differences in their thresholds are more similar to experimental observations [12]. We modified release rates from IHC-HT SGN and IHC-MT SGN synapses so that HT and MT fibers are not responsive to sound stimuli lower than 30dB SPL and 15dB SPL, respectively. Our results (S1B–S1E Fig) show that modifying thresholds didn’t change the CAP properties of different scenarios significantly.

In the myelinopathy simulations, we varied the length of the initial unmyelinated segment *L*_*u*_ keeping a constant channel density (Fig 5) and varied the length of the heminode *L*_*h*_ keeping constant channel numbers (Fig 6). Results showed similar effects on SGN fiber activity, i.e. the populations with the same combined lengths *L*_*u*_+*L*_*h*_ exhibited the same behavior. As evidence is lacking on how ion channel distributions might be affected by the disruption of myelination patterns, we also simulated cases where *L*_*u*_ increases with constant channel number (S2 Fig) and *L*_*h*_ increases with constant channel density (S3 Fig). Results showed that spreading the same number of channels over an increased *L*_*u*_ (S2 Fig), rather than increasing the number by keeping the channel density constant (Fig 5), didn’t change the SGN fiber activity significantly. In contrast, varying *L*_*h*_ while keeping the heminode channel density constant, i.e., increasing the number of channels for larger *L*_*h*_, increased the *L*_*h*_ value associated with the loss of spike generation up to 4 μm, compared to 2 μm when channel number was kept constant (Fig 6). To conclude, any of these scenarios results in qualitatively similar SGN fiber activity patterns and they might only affect the *L*_*u*_ and *L*_*h*_ lengths at which loss of spike generation leads to an abrupt drop in the CAP peak.

To better understand the effects of myelinopathy on SGN spike generation, we additionally analyzed the outcome of one vesicle release event to a single SGN fiber. As described in the Methods section, SGN response to vesicle release was simulated by applying a brief external current pulse to the peripheral end of the SGN fibers. We calculated the time difference between a spike of one SGN fiber and the preceding release event, which we define as delay, for various conductances (*A*) (S4 Fig). These results provided evidence that increasing *L*_*u*_ leads to longer spike delays, up to a critical value of *L*_*u*_ for all values of *A*. For *L*_*u*_ values higher than the critical value, a single release event does not result in a spike on an SGN fiber. Results showed that this critical value is larger for higher values of *A*. However, different *I*_*app*_ characteristics exhibited qualitatively similar trends, meaning that synaptic efficacy wouldn’t affect our results qualitatively. We additionally investigated the population outcome of vesicle release events to the SGN fibers. We thus calculated the probability that release events result in corresponding spikes for various conductances *A* of the external current pulse *I*_*app*_ for increasing values of *L*_*u*_ (S5A Fig). For simulated 70dB SPL stimuli, higher *I*_*app*_ conductances increased spike probability for larger *L*_*u*_ values, leading to increases in the *L*_*u*_ values at which spike generation was affected. If *L*_*u*_ exceeded a critical value, the probability of spike generation decreased significantly, due to the fact that one release event is not enough to generate a spike in an SGN fiber with an *L*_*u*_ higher than this critical value, as shown in S4 Fig. These results show that this *L*_*u*_ critical value required for spike generation depends on IHC-SGN synaptic efficacy.

To analyze the effect of sound level on SGN fiber spike probability, we ran simulations for all sound levels keeping the conductance of *I*_*app*_, *A*, fixed at the default value (*A* = 0.12nS, solid black rectangle in S5A Fig). As described in the Methods section, increasing sound level was simulated by [12,13,21] a higher IHC release rate, i.e. higher frequencies of external current pulse applications to SGN fibers. For this *A* value, spike generation was affected for *L*_*u*_>11.6 μm as evident in the results shown in Fig 5. For SGN fibers with *L*_*u*_ ≤ 11.6 μm, spike probabilities were higher than 40% for all sound levels (S5B Fig). However, spike probabilities decreased gradually with higher sound levels due to the inability of the fibers to respond to high frequency stimulation. This means, SGN fibers cannot fire with a higher frequency due to the saturation of their spike rate, resulting in decreased spike probabilities. For SGN fibers with *L*_*u*_>11.6 μm, spike probability was very low reflecting loss of spike generation, but it increased slightly with increasing sound level, as high frequency stimulation facilitated spike generation due to temporal summation. Results for heterogeneous *L*_*u*_ values between 10 and 20 μm showed low spike probabilities (~20%) as compared to homogeneous *L*_*u*_ values of 10 μm, for all sound levels.

Lastly, to analyze effects of myelinopathy on SGN spike latency, we averaged the time differences between each spike and the preceding release event causing the spike for populations of SGN fibers with varied homogeneous *L*_*u*_ values and varied sound levels (S5C Fig). The populations with *L*_*u*_>11.6 μm were not included since spikes were not reliably generated and for the heterogeneous population, the fibers with *L*_*u*_>11.6 μm were ignored. The homogeneous populations showed increased latencies with increasing *L*_*u*_ and the heterogeneous population’s latencies were similar to the *L*_*u*_ = 11 μm case. Latencies decreased with increasing sound levels. However, standard deviations of spike latencies increased with sound level, presumably reflecting higher variability in spike response to higher frequency stimulation (S5D Fig). Additionally, the population with heterogeneous *L*_*u*_ values showed higher standard deviations for all sound levels than the putative normal case with *L*_*u*_ = 10 μm. This increase in spike timing variability is responsible for increases in the width of the cumulative CAP for the heterogeneous population shown in Fig 5.

In conclusion, our model results showed that HHL deficits in sound-evoked AN activity due to myelinopathy could be caused by not only loss of SGN spike activity, as in synaptopathy, but also disruption of spike timing and synchronization across a population of SGN fibers. Furthermore, these results can be obtained with simpler models of inner hair cell release, without taking the nonlinear dynamics of cochlear sound processing into account, illustrating the significance of the SGN fiber organization on the observed deficits [37]. Illumination of the underlying differences on neural activity caused by myelinopathy and synaptopathy may be useful for understanding the effects on central auditory processing and resulting perceptual deficits. In this way, the model framework provides a testbed for the development and testing of predictions for potential interventions for the mitigation and treatment of HHL.

## Supporting information

S1 Fig Modifying the thresholds of MT and HT fibers does not have a significant effect on CAP features for different HHL scenarios. (A)The rate-intensity curves for different fiber types (see Fig 2E for unmodified curves) are modified to have a more clear distinction between the activity thresholds of different fiber types, as in Fig 3 of [12]. The release rates of IHC-HT SGN synapses and IHC-MT SGN synapses are set to zero for sound levels less than 30dB SPL and 15 dB SPLs, respectively. Panels B-E are simulated based on these release rates. (B) Sound-evoked CAPs of SGN fiber populations with different myelinopathy and synaptopathy scenarios at 70dB SPL, averaged over 50 simulations (dashed lines correspond to the peaks of each CAP, labeled with the same colors as the CAPs). Combined synaptopathy and myelinopathy (Case 3) showed additive effects on the decrease in CAP peak amplitude, but not on the increase in CAP peak latency (compare to Cases 1 and 2). Combined different myelinopathies showed additive effects on both CAP peak amplitude and latency (compare Cases 2 and 4). (C) Comparison of average CAP measures for different myelinopathy and synaptopathy cases relative to normal, and between cases at 70 dB SPL (*p<0.05, **p<0.01, #p<0.001). Normalized CAP amplitudes (D) and CAP latencies (D) for different myelinopathy and synaptopathy cases for various sound levels, averaged over 50 simulations. Shaded areas correspond to the standard error of the mean.
(TIF)

S2 Fig Keeping constant channel number as length of unmyelinated segment, *L*_*u*_, is increased leads to similar effects on cumulative CAP as keeping constant channel density. (A)Sound-evoked CAPs of SGN fiber populations with varied *L*_*u*_ at 70dB SPL, averaged over 50 simulations (dashed lines correspond to the peaks of each CAP, labeled with the same colors as the CAPs). The number of membrane ionic channels was kept fixed at the values for normal *L*_*u*_ (*L*_*u*_ = 10 μm). Decreases in peak amplitude and increases in peak latency are similar for populations with *L*_*u*_ > 11 μm (compare to Fig 5). (B) Comparison of CAP measures relative to normal *L*_*u*_ (*L*_*u*_ = 10 μm) of each population at 70 dB SPL (*p<0.05, **p<0.01, #p<0.001). Normalized CAP amplitudes (C) and CAP latencies (D) for various sound levels, averaged over 50 simulations. Shaded areas correspond to the standard error of the mean.
(TIF)

S3 Fig Maintaining channel density at the heminode as its length, *L*_*h*_, is varied reduces effects on cumulative CAP compared to keeping constant channel number. (A) Sound-evoked CAPs of SGN fiber populations with varied *L*_*h*_ at 70dB SPL, averaged over 50 simulations (dashed lines correspond to the peaks of each CAP, labeled with the same colors as the CAPs). Densities of membrane ionic channels were kept constant at the values for normal *L*_*h*_ (*L*_*h*_ = 1 μm). (B) Comparison of CAP measures relative to normal *L*_*h*_ (*L*_*h*_ = 1 μm) for each population at 70 dB SPL (*p<0.05, **p<0.01, #p<0.001). Decreases in amplitude and increases in latency of CAP peaks are more obvious for populations with *L*_*h*_ > 4 μm (compare to Fig 6). Normalized CAP amplitudes (C) and CAP latencies (D) for various sound levels, averaged over 50 simulations. Shaded areas correspond to the standard error of the mean.
(TIF)

S4 Fig The conductance of Iapp (A) and the Lu value of SGN fibers determine the time difference between a spike and a release preceding the spike (delay). External current pulses with varying conductances, A, are applied to the peripheral end of SGN fibers to simulate response to a vesicle release event, and the time difference between the release and the resulting spike is calculated for single SGN fibers with varying Lu. Red curve represents our default A value for simulating release responses, unless otherwise stated.
(TIF)

S5 Fig Myelinopathy results in a significantly reduced spike probability and increased latency after a release event. (A) The probability that simulated IHC-SGN synaptic vesicle release events result in spike generation at the heminodes of postsynaptic SGN fibers was calculated for various SGN fiber populations at 70dB SPL, averaged over 50 simulations. The conductance *A* of external current pulses (*I*_*app*_) applied at the beginning of *L*_*u*_, representing IHC-SGN vesicle release, was varied between 0.10nS and 0.14nS. The threshold *L*_*u*_, where abrupt drop of spike probability occurs, increases with increasing *A*. Panels (B)-(D) and all other results in the paper were obtained with *A* = 0.12nS. (B) Spike probabilities for SGN fiber populations with different homogeneous *L*_*u*_ values in response to different sound levels exhibit an abrupt drop when *L*_*u*_ ≥11.7 μm for all sound levels. (C) The average latency after each release event of spikes across SGN fiber populations, averaged over 50 simulations, increases for longer *L*_*u*_. (D) Standard deviations of spike latencies of SGN fiber populations, averaged over 50 simulations, increase with sound level. The heterogeneous population (10 μm ≤ *L*_*u*_ ≤ 20 μm) has higher standard deviation than the putative control case (*L*_*u*_ = 10 μm) for every sound level. Since fibers with *L*_*u*_ >11.6 μm do not fire in response to single release events, they are not shown in panels (C) and (D).
(TIF)
