# Correction: Contrasting mechanisms for hidden hearing loss: Synaptopathy vs myelin defects

**DOI:** 10.1371/journal.pcbi.1008910

**Published:** 2021-04-07

**Authors:** Maral Budak, Karl Grosh, Aritra Sasmal, Gabriel Corfas, Michal Zochowski, Victoria Booth

The funding statement for this article should read as follows:

“This work was supported by National Institutes of Health—National Institute on Deafness and Other Communication Disorders: NIDCD R01DC004820 (G.C.), National Institutes of Health—National Institute on Deafness and Other Communication Disorders NIDCD R01DC018500 (G.C.) and National Institute of Biomedical Imaging and Bioengineering: NIBIB R01EB018297 (M.Z., V.B.). The funders had no role in study design, data collection and analysis, decision to publish, or preparation of the manuscript.”
