# Supplementary figures and images for: Contrasting mechanisms for hidden hearing loss: Synaptopathy vs myelin defects
Source: PLoS Comput Biol. 2021 Jan 22;17(1):e1008499. doi: 10.1371/journal.pcbi.1008499 (PMC7857583; doi:10.1371/journal.pcbi.1008499)

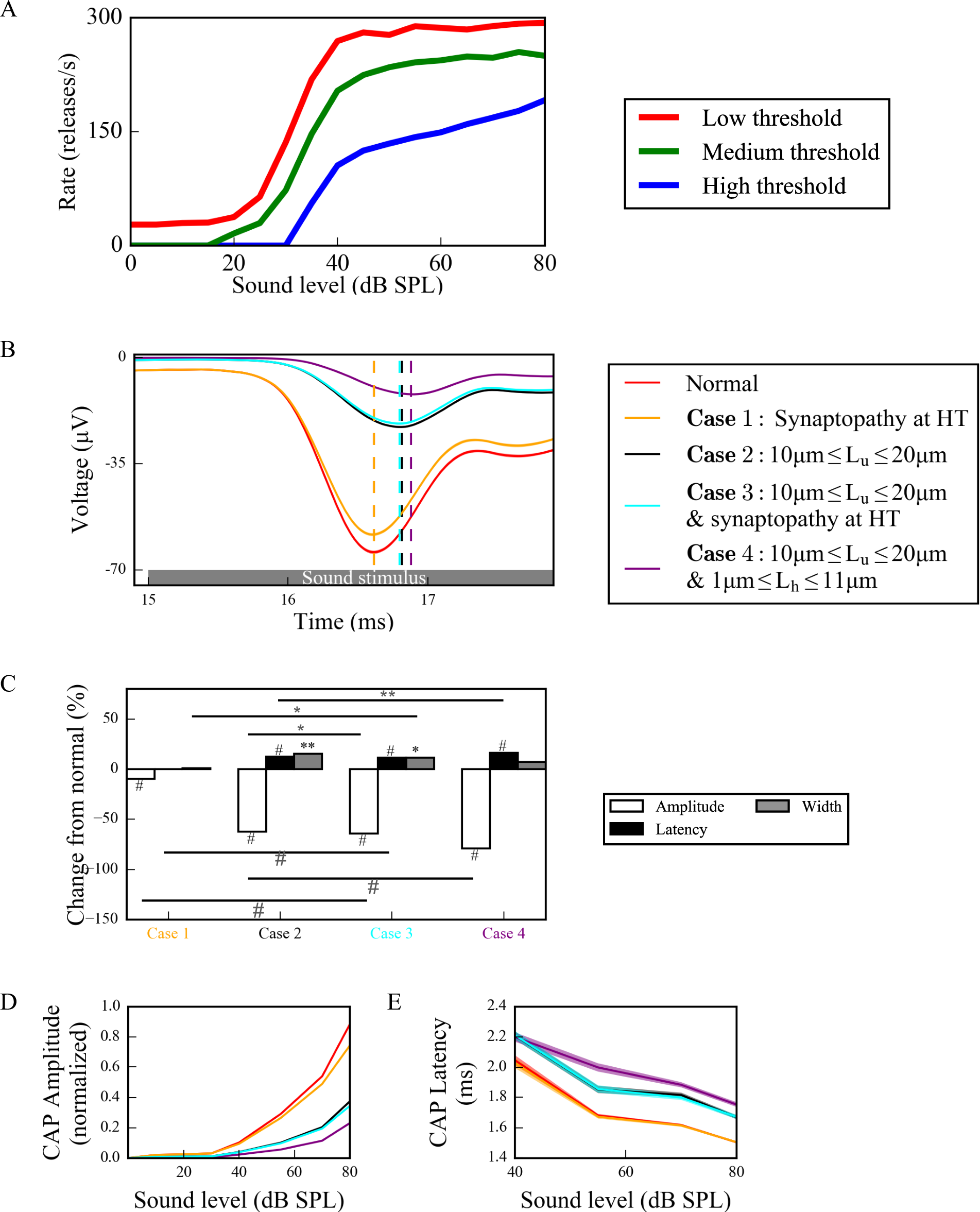

Supplement: S1 Fig — (A)The rate-intensity curves for different fiber types (see Fig 2E for unmodified curves) are modified to have a more clear distinction between the activity thresholds of different fiber types, as in Fig 3 of [12]. The release rates of IHC-HT SGN synapses and IHC-MT SGN synapses are set to zero for sound levels less than 30dB SPL and 15 dB SPLs, respectively. Panels B-E are simulated based on these release rates. (B) Sound-evoked CAPs of SGN fiber populations with different myelinopathy and synaptopathy scenarios at 70dB SPL, averaged over 50 simulations (dashed lines correspond to the peaks of each CAP, labeled with the same colors as the CAPs). Combined synaptopathy and myelinopathy (Case 3) showed additive effects on the decrease in CAP peak amplitude, but not on the increase in CAP peak latency (compare to Cases 1 and 2). Combined different myelinopathies showed additive effects on both CAP peak amplitude and latency (compare Cases 2 and 4). (C) Comparison of average CAP measures for different myelinopathy and synaptopathy cases relative to normal, and between cases at 70 dB SPL (*p<0.05, **p<0.01, #p<0.001). Normalized CAP amplitudes (D) and CAP latencies (D) for different myelinopathy and synaptopathy cases for various sound levels, averaged over 50 simulations. Shaded areas correspond to the standard error of the mean. (TIF) [file pcbi.1008499.s001.tif]

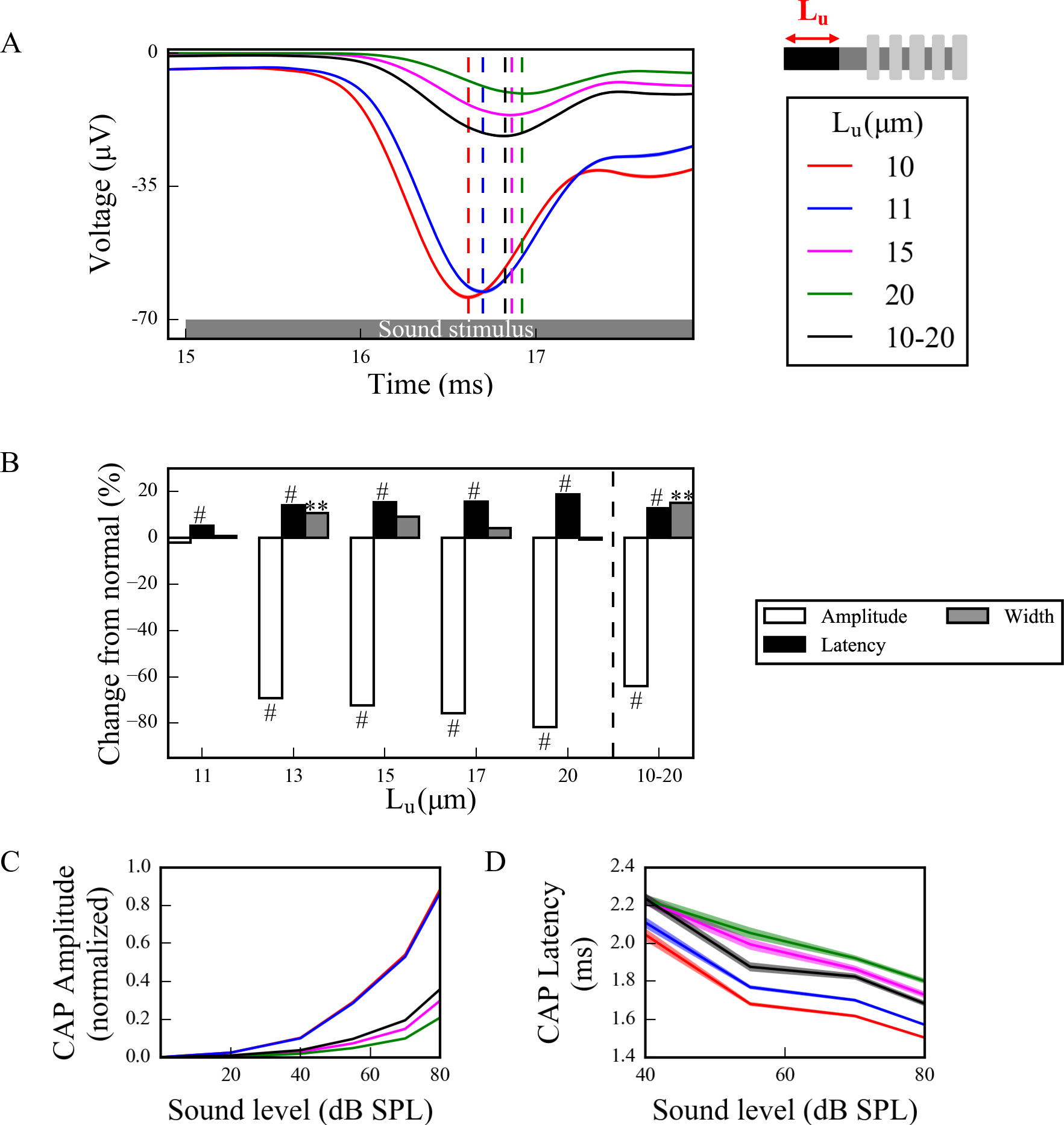

Supplement: S2 Fig — (A)Sound-evoked CAPs of SGN fiber populations with varied Lu at 70dB SPL, averaged over 50 simulations (dashed lines correspond to the peaks of each CAP, labeled with the same colors as the CAPs). The number of membrane ionic channels was kept fixed at the values for normal Lu (Lu = 10 μm). Decreases in peak amplitude and increases in peak latency are similar for populations with Lu > 11 μm (compare to Fig 5). (B) Comparison of CAP measures relative to normal Lu (Lu = 10 μm) of each population at 70 dB SPL (*p<0.05, **p<0.01, #p<0.001). Normalized CAP amplitudes (C) and CAP latencies (D) for various sound levels, averaged over 50 simulations. Shaded areas correspond to the standard error of the mean. (TIF) [file pcbi.1008499.s002.tif]

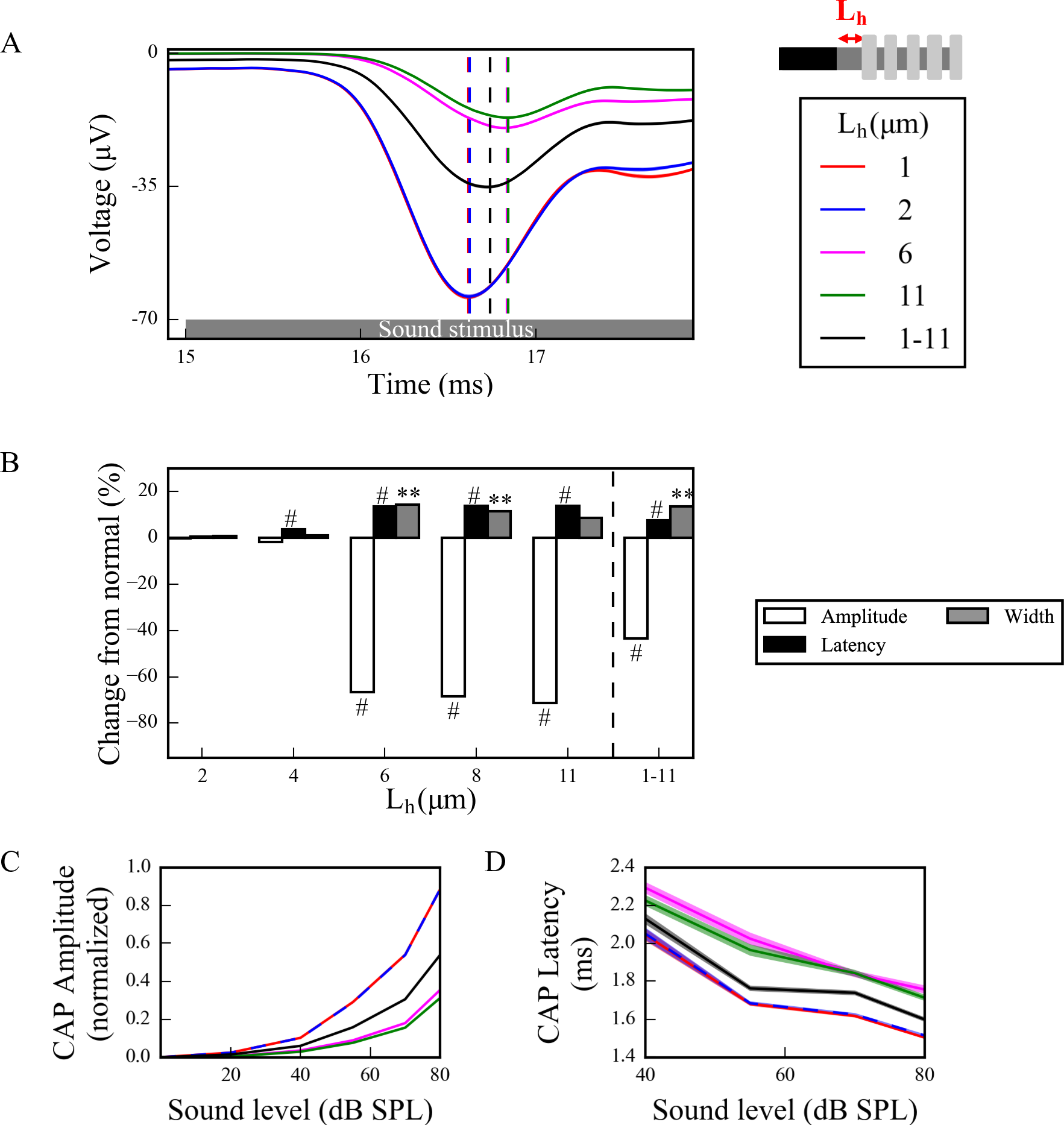

Supplement: S3 Fig — (A) Sound-evoked CAPs of SGN fiber populations with varied Lh at 70dB SPL, averaged over 50 simulations (dashed lines correspond to the peaks of each CAP, labeled with the same colors as the CAPs). Densities of membrane ionic channels were kept constant at the values for normal Lh (Lh = 1 μm). (B) Comparison of CAP measures relative to normal Lh (Lh = 1 μm) for each population at 70 dB SPL (*p<0.05, **p<0.01, #p<0.001). Decreases in amplitude and increases in latency of CAP peaks are more obvious for populations with Lh > 4 μm (compare to Fig 6). Normalized CAP amplitudes (C) and CAP latencies (D) for various sound levels, averaged over 50 simulations. Shaded areas correspond to the standard error of the mean. (TIF) [file pcbi.1008499.s003.tif]

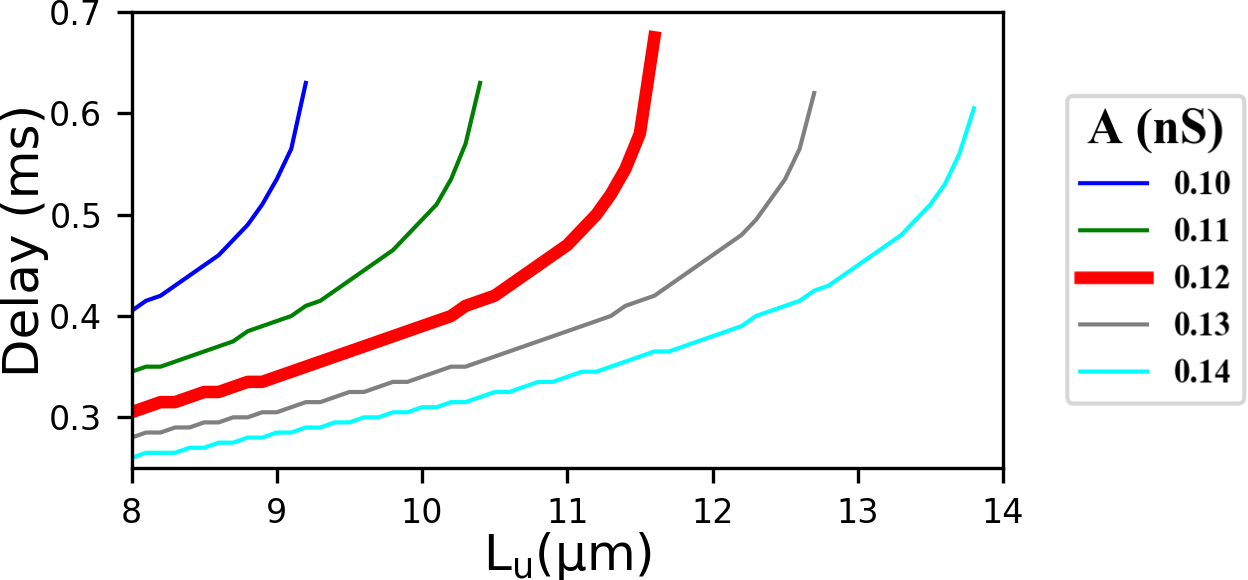

Supplement: S4 Fig — The conductance of Iapp (A) and the Lu value of SGN fibers determine the time difference between a spike and a release preceding the spike (delay). External current pulses with varying conductances, A, are applied to the peripheral end of SGN fibers to simulate response to a vesicle release event, and the time difference between the release and the resulting spike is calculated for single SGN fibers with varying Lu. Red curve represents our default A value for simulating release responses, unless otherwise stated. (TIF) [file pcbi.1008499.s004.tif]

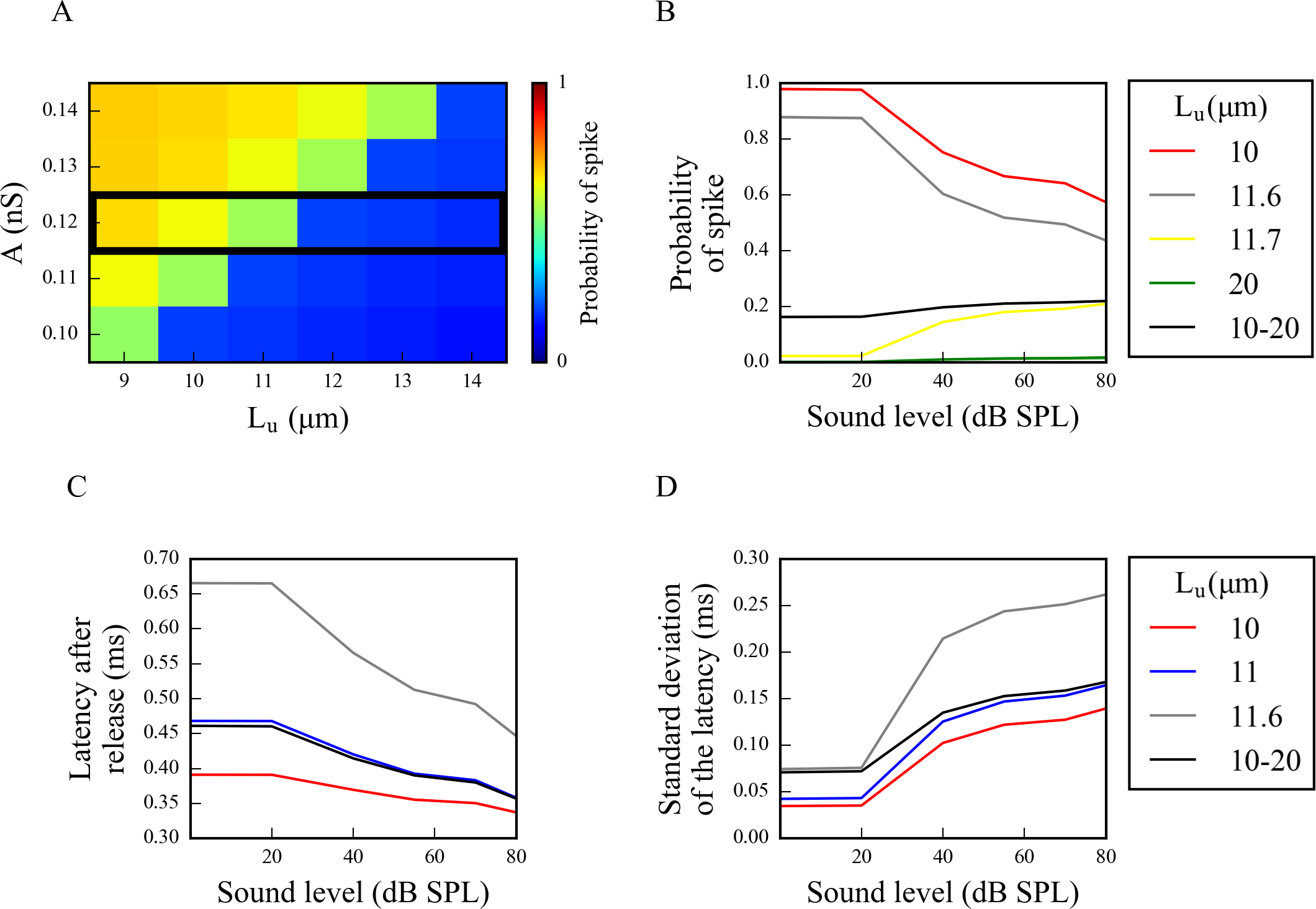

Supplement: S5 Fig — (A) The probability that simulated IHC-SGN synaptic vesicle release events result in spike generation at the heminodes of postsynaptic SGN fibers was calculated for various SGN fiber populations at 70dB SPL, averaged over 50 simulations. The conductance A of external current pulses (Iapp) applied at the beginning of Lu, representing IHC-SGN vesicle release, was varied between 0.10nS and 0.14nS. The threshold Lu, where abrupt drop of spike probability occurs, increases with increasing A. Panels (B)-(D) and all other results in the paper were obtained with A = 0.12nS. (B) Spike probabilities for SGN fiber populations with different homogeneous Lu values in response to different sound levels exhibit an abrupt drop when Lu ≥11.7 μm for all sound levels. (C) The average latency after each release event of spikes across SGN fiber populations, averaged over 50 simulations, increases for longer Lu. (D) Standard deviations of spike latencies of SGN fiber populations, averaged over 50 simulations, increase with sound level. The heterogeneous population (10 μm ≤ Lu ≤ 20 μm) has higher standard deviation than the putative control case (Lu = 10 μm) for every sound level. Since fibers with Lu >11.6 μm do not fire in response to single release events, they are not shown in panels (C) and (D). (TIF) [file pcbi.1008499.s005.tif]
